# Efficacy of the Adapted Cycles Intervention Program (PROCICLOS-A) and the influence of severity on the performance of children with Speech Sound Disorders

**DOI:** 10.1590/2317-1782/e20240215en

**Published:** 2025-07-28

**Authors:** Mayra Alexandra Misugi, Daniel Gomes dos Santos, Carolina Kuntz Ayub, Haydée Fiszbein Wertzner

**Affiliations:** 1 Programa de Pós-graduação em Ciências da Reabilitação, Departamento de Fisioterapia, Fonoaudiologia e Terapia Ocupacional, Faculdade de Medicina, Universidade de São Paulo – USP - São Paulo (SP), Brasil.; 2 Curso de Fonoaudiologia, Departamento de Fisioterapia, Fonoaudiologia e Terapia Ocupacional, Faculdade de Medicina da Universidade de São Paulo – USP - São Paulo (SP), Brasil.; 3 Departamento de Fisioterapia, Fonoaudiologia e Terapia Ocupacional, Faculdade de Medicina, Universidade de São Paulo – USP - São Paulo (SP), Brasil.

**Keywords:** Speech Therapy, Speech, Child, Speech Sound Disorders, Intervention

## Abstract

**Purpose:**

To verify the efficacy of PROCICLOS-A in children with SSD, according to the weekly frequency of sessions, and the severity's influence on the sessions' performance.

**Methods:**

Eight children with SSD participated, both sexes, ages between 5:03 and 7:07 years, divided into two groups: C1, once a week; C2, twice a week. The efficacy of PROCICLOS-A was verified in C1 and C2 by analyzing variables from the phonology tests of the ABFW assessment across three evaluation moments. The performance in the sessions was obtained through scoring the activities.

**Results:**

All measures significantly differed, indicating improved performance in the evaluations after the intervention. Regarding the influence of the frequency of the sessions, there was no significant difference between C1 and C2 in the three evaluation moments. Blocks of sessions analyzed the performance in the sessions: A (sessions 1, 4, 7, 10), B (sessions 2, 5, 8, 11), C (sessions 3, 6, 9, 12). The analysis revealed lower performance during the sessions of Block A, while Blocks B and C demonstrated better performance.

**Conclusion:**

The study provided evidence for the efficacy of PROCICLOS-A, regardless of the dosage and frequency of sessions. It suggests that maintaining the cumulative intensity of the intervention is vital, indicating that the total number of intervention hours may have a greater impact than the number of sessions held per week. There was an improvement in each child's performance throughout the program, regardless of the target sound and severity at the beginning of the intervention.

## INTRODUCTION

Speech sound disorder (SSD) refers to speech difficulties that can manifest in different combinations of phonological, perceptual, and motor production aspects. The SSD addressed in this study is of unknown cause, with its main characteristic being phonological impairment, characterized by a cognitive-linguistic difficulty involving the phonological rules of the language^(1-3)^.

Children with phonological impairment and SSD have difficulties in the phonological representations of speech segments, phonotactic structure, and/or stress patterns, observed in how they use, represent, and mentally organize the phonological system of their language^(3)^. They may also have difficulties in speech perception skills and/or phonological processing, including phonological awareness skills^(4,5)^.

Combined manifestations in the speech of children with SSD are quite variable, characterizing heterogeneity among them. Therefore, severity and speech intelligibility are variable, requiring Speech-Language Pathologist (SLP) evaluation with tests that allow a detailed description of the child's speech and indicate their strengths and weaknesses.

The literature has proposed some measures to describe the severity of SSD and enable comparisons between the speech of different children. These include the Percentage of Consonants Correct (PCC)^(6)^, the Percentage of Consonants Correct-Revised (PCC-R)^(7)^, and the Phonological Density Index (PDI)^(8)^. These three severity measures have been used to indicate the efficacy of intervention approaches in SSD^(9-11)^.

### SSD intervention

It is recommended that intervention in SSD occurs as early as possible, due to the risk of children with SSD having difficulties in learning to read and write and in literacy, besides academic, social, and vocational failure. Such difficulties can have long-term consequences, preventing children from reaching their full potential^(3,12-15)^.

Some approaches to SSD are generally more focused on the production of speech sounds, cognitive-linguistic (phonological) aspects, and/or perception, each of them being applicable to a type of SSD. Considering this great division between approaches, there are several proposals for each of them and an attempt to classify and better understand the elements that make up such interventions^(13,16,17)^. Selecting the intervention approach for SSD of unknown cause depends on what the SLP assessment has found.

Another essential factor to be considered in SSD intervention is the intensity and frequency required to obtain effective results when applying a therapeutic approach. Studies have addressed this issue by exploring the relationship between intensive sound production and the regularity of the intervention. Thus, the weekly frequency of the sessions and the dose of stimuli offered in the sessions represent the intensity of the intervention^(9,18-20)^.

The literature highlights that, in general, more favorable prognoses for children are associated with a therapeutic approach characterized by greater frequency and intensity^(9,18,19,21,22)^. Thus, it is essential to understand the importance not only of effective therapeutic approaches but also the need for an intensive and consistent approach to optimize children’s speech development results.

A systematic review study verified the efficacy and efficiency of the available approaches and suggested that some of them lack details and clarification, as they are ambiguous regarding their application and the way they are delivered to children by SLP^(17)^.

They also highlight the need for randomized studies that investigate the efficacy, frequency of sessions, and dose of stimuli per session.

### Cycles approach

The cycles approach, proposed by Hodson and Paden^(23)^ stands out among those that seek to intervene in SSD. It is based on the interaction of cognitive-linguistic, perceptive, and speech production processes. In this approach, intervention priorities must be set considering the child’s phonological processes in the initial assessment and the stimulability of the sounds absent from their phonetic inventory.

The intervention is developed in cycles, with each one corresponding to the period when the phonological processes that most affect the intelligibility of the child's speech are addressed. Each target sound of the selected phonological process is addressed for 60 minutes and then switched to another target sound. Thus, several sounds are addressed in a cycle. Moreover, specific activities are proposed using semantic, tactile-kinesthetic, auditory, and visual cues, guiding the child through strategies that favor the development of speech and language skills.

A study by Rudolph and Wendt^(24)^ shows that the cycles approach proposed by Hodson and Paden^(23)^ is effective, as it combines an appropriate selection of each child's phonological processes, treated in a cyclical format, through activities that work on auditory input and practice in producing the target sound. Thus, the approach improves the exchanges in the child's speech, even generalizing unworked sounds. Furthermore, the study suggests that it is possible to apply variations to the approach, such as intensity, strategies to work on the target sound, and the order in which each target sound is addressed, as ways to achieve better performances in children.

Another study by Cabbage et al.^(25)^, which investigates the choice of therapists in view of the different therapeutic approaches available, shows that among the most diverse interventions, those involving a traditional articulatory approach, such as the Hodson and Paden Cycles approach^(23)^, are the most chosen by SLP pathologists. The results showed that not only did the interviewees use the Cycles approach for treatment, but they also tended to combine it with other approaches, according to each case and need.

Based on the original Cycles proposal by Hodson and Paden^(23)^, an adaptation for Brazilian Portuguese speakers was developed by Wertzner and Pagan-Neves^(26)^. In the Adapted Cycles approach, the authors propose 7-session cycles, in which the phonological process and the stimulable target sound are selected based on the initial assessment. Sessions 1 to 3 use auditory bombardment activities of the target sound, phonemic placement, articulatory reinforcement of the target sound, auditory discrimination activities, and activities with minimal pairs involving the chosen target sound. Sessions 4 to 7 add metaphonology activities; also, at the end of the 7^th^ session, the verification test is applied to observe the generalization that has occurred^(26)^.

This study presents the Adapted Cycles Program (PROCICLOS-A, in Portuguese), in which each cycle has 12 sessions. Two phonological processes and a total of four target sounds are addressed throughout the sessions. The child's performance in each activity of the sessions is scored, allowing us to observe the easiest and most difficult target sound and the skills that represent the greatest challenge for the child. It aims to answer the following questions: “Do children who obtained lower values ​​in the ABFW Phonology measures in the initial assessment perform better in the PROCICLOS-A sessions? Does the number of sessions per week affect the children's improvement?”.

The study aimed to verify the efficacy of PROCICLOS-A in children with SSD according to the weekly frequency of sessions and the influence of severity on session performance.

## METHODS

### Participants

This prospective, experimental, randomized study was approved by the Research Ethics Committee (CAAE 87068318.2.0000.0065). The parents/guardians signed an informed consent form, and the children signed an assent form. This study is part of intervention research, in which children are randomly assigned to three different intervention programs with different weekly frequencies.

Randomization was performed electronically, using the “random” function of the Excel program, by the last author of the study, and was designed to ensure a balanced distribution among the three interventions. Eight children participated in this study and were randomly allocated to PROCICLOS-A in two different groups, according to the frequency of the sessions: the first group, with four children, underwent the intervention once a week (C1); the second group, also with four children, underwent the intervention twice a week (C2).

The speech-language diagnostic assessment was performed in a research laboratory linked to a university, using the following protocols: ABFW Child Language Test in the areas of phonology, vocabulary, fluency, and pragmatics^(27)^, Phonological Sensitivity Test – Auditory (TSF-A)^(28,29)^, Comprehensive Test of Phonological Processing (CTOPP – Rapid Naming)^(30)^, Auditory Discrimination (an unpublished protocol developed in the research laboratory, which includes contrasts of point, mode, and voicing, in one and two-syllable words), Speech Inconsistency Test^(31)^, Orofacial Myofunctional Evaluation with Scores (OMES)^(32)^, and the Speech Sound Stimulability Test (TESF)^(33)^.

The eight children with SSD, of both sexes, were aged 5:3 to 7:7 years. Inclusion criteria were obtaining below 93.4%^(34)^ PCC-R^(7)^ in the initial assessment with the naming test of the ABFW Phonology Child Language Test in phonology, vocabulary, fluency, and pragmatics (ABFW Phonology)^(35)^; manifesting at least one phonological process with a minimum occurrence of 25% in one of the two ABFW Phonology tests; having an audiological evaluation within normal limits (below 20 dB at 500, 1000, 2000, and 4000 Hz); having a non-verbal IQ within normal limits^(36)^, verified by a professional psychologist; having Brazilian Portuguese as their native language.

### Procedures

#### Baseline assessment and reassessments

The children were assessed at three different moments. The first one, before beginning the therapeutic intervention, was called the baseline assessment (A1); the second assessment (A2) occurred after applying PROCICLOS-A; and the third assessment (A3) was carried out 6 weeks after the second assessment, in which the children were left without intervention. The baseline (A1) and reassessments considered the ABFW^(35)^ phonology naming and imitation tests, for which the following measures were calculated: PCC^(6)^, PCC-R^(7)^, PDI^(8)^, and the number of phonological processes (NPP) with occurrence > 25%. The assessments were recorded on video and analyzed by two SLP pathologists, with 85% agreement. The results of the evaluations were analyzed to verify the efficacy of PROCICLOS-A.

Each child’s diagnostic assessment and reassessments throughout and at the end of the intervention programs were performed by a SLP pathologist, a graduate student, who was blind to the study intervention procedure. Likewise, the SLP pathologist who applied PROCICLOS-A was blind to the assessment, and the senior researcher (fourth author) selected the target sounds to be addressed in the sessions.

The criteria for selecting the phonological processes and target sounds were preferably phonological processes with occurrence > 25%, no longer expected for the child's age, and which compromised their speech intelligibility. The stimulability of the sounds was also considered, choosing stimulable ones.

### PROCICLOS-A

PROCICLOS-A^(37)^ is an adaptation of the Adapted Cycles Approach^(26)^ which, in turn, was developed as a modification of the Hodson and Paden Cycles approach^(23)^. PROCICLOS-A has 12 intervention sessions, with an average duration of 50 minutes, which aim to develop auditory perception, proprioception of articulatory movements, phonological rules, and phonological awareness.

**Figure 1:**
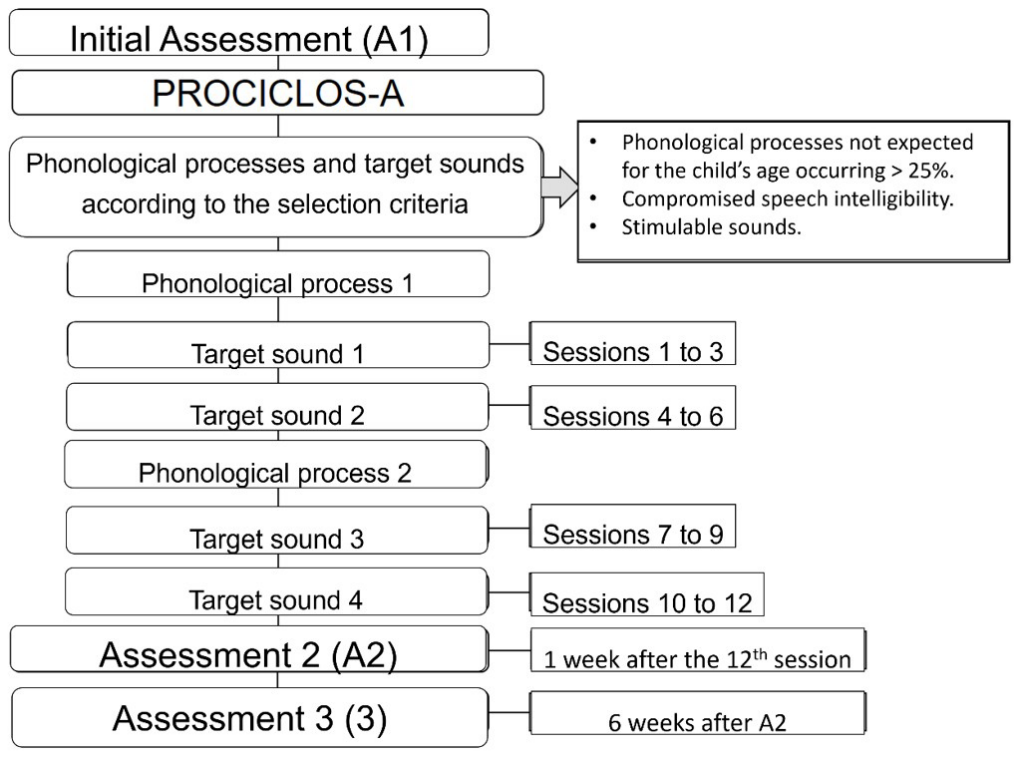
PROCICLOS-A approach outline

As PROCICLOS-A in the Cycles approach^(23)^ considers that mastery of sounds and phonological rules is acquired gradually, SSD intervention should gradually expose the child to speech sounds. Thus, new target sounds are introduced in the treatment before the previous ones are mastered. Two phonological processes and two target sounds in each of them are selected to be worked on during the 12 sessions. The criteria for selecting phonological processes include those that most compromise speech intelligibility and that are in accordance with what is expected for the child's age. Stimulable target sounds are chosen for each phonological process. Sessions 1 to 6 address the two target sounds of the first phonological process, with sessions 1 to 3 referring to the first, and sessions 4 to 6 to the second process. Sessions 7 to 12 deal with the second phonological process, with the first target sound being addressed in sessions 7 to 9, and the second target sound in sessions 10 to 12, as shown in Figure 1.

**Caption:** Block: A (sessions 1, 4, 7, and 10), B (sessions 2, 5, 8, and 11), and C (sessions 3, 6, 9, and 12)

Six types of activities are developed in each of the 12 SLP intervention sessions, covering the target sounds selected for each session. The selected activities were Auditory Bombardment (at the beginning and end of the session); Placement of the Target Sound; Recognition of the Target Sound and Auditory Discrimination with Minimal Pairs; Activities with Minimal Pairs for Understanding the Rule; Word Training with the Target Sound in Initial, Medial, and Final Position; and Phonological Awareness Activities (Chart 1). The activities were planned for the sessions to last an average of 50 minutes and for the child to produce the target sound at least 100 times during each session.

**Chart 1:** PROCICLOS-A session activities

1. Auditory Bombardment	The speech-language-hearing therapist slowly reads the disyllabic words that begin with the target sounds of the process being worked on. The child must listen carefully to these words. Average duration of the activity: 1 minute.
2. Presentation and Articulatory Production of the Target Sound	This activity aims to help the child produce sounds through multimodal facilitating cues – i.e., auditory, visual, and tactile cues. Average duration of the activity: 10 minutes.
3. A) Auditory Recognition; B) Auditory Discrimination	In Auditory Recognition, the child must recognize the target sound worked on whenever they hear it. In Auditory Discrimination, they must identify which pair is the same and which pair is different. Average duration of the activities: 5 minutes.
4. Understanding the Rule with Minimum Pairs	The activity aims to help children understand and use phonological rules. For each target sound of a phonological process, 3 to 5 minimal pairs were selected by minimum opposition or maximum opposition. Average activity time: 15 to 20 minutes.
5. Training on words with the target sound in initial, medial, and final position	Strategies are used to work on the correct production of the target sound when in initial, medial, and final syllables. Average strategy time: 15 to 20 minutes.
6. Phonological Awareness	The goal is to stimulate phonological awareness through rhyming, alliteration, and syllabic segmentation skills. Average duration of the strategy: 10 minutes.
7. Auditory Bombardment	The speech-language-hearing therapist slowly reads the disyllabic words that begin with the target sounds of the process being worked on. The child must listen carefully to these words. Average duration of the activity: 1 minute.

Different strategies were developed for each activity, and their analysis in the 12 sessions considered their performance by block of sessions: A, B, and C. Block A corresponds to sessions numbers 1, 4, 7, and 10, referring to the introduction of a new target sound. Block B corresponds to sessions numbers 2, 5, 8, and 11, and refers to the medial sessions of each target sound. Block C corresponds to sessions numbers 3, 6, 9, and 12, and refers to the last sessions worked with each chosen target sound.

### Session Performance Scoring

A scoring system was developed to analyze performance in each of the 12 sessions and in the PROCICLOS-A activities. Hence, 2 points are assigned for correct answers, 1 point for partially correct answers, and 0 for errors. Answers were partially correct when the target sound was produced with distortion, or correct answers were obtained on the second attempt at production. Activities 2, 3, 4, 5, and 6 are scored (Chart 1). Since the stimuli for each phonological process and target sound vary, the total value expected in each session also varies.

Excel^®^ spreadsheets were created for each target sound to record the children's performance, indicating the expected values ​​for each activity, and recording the results. Each child's score per session in this study was assigned by the first two authors, calculating their agreement, which remained above 80% for all sessions of the eight children in the study.

### Statistical analysis

Descriptive data analysis included calculation of measures of central tendency and dispersion. The Mann-Whitney U test was used for intergroup comparisons, and Friedman's univariate analysis of variance (ANOVA) for intragroup comparisons, with post hoc analysis using the Wilcoxon signed-rank test with Bonferroni correction for multiple comparisons. Correlation analyses were performed using Spearman's correlation test. Due to the low sample size, all hypotheses were tested using nonparametric procedures; in addition to the significance level set at 5% (p ≤ 0.05), the effect size was also measured by calculating the r coefficient^(38)^ to complement the interpretation of the results. To interpret the effect size (ES), it is suggested to use the classification proposed by Cohen^(39)^: small (between |0.200| and |0.499|); medium (between |0.500| and |0.799|); and large (above |0.800|).

## RESULTS

The study results include verification of the efficacy of PROCICLOS-A, comparing the three evaluation moments for each of the groups C1 and C2, the analysis of the influence of the frequency dose per session, and the performance in the PROCICLOS-A sessions.

Chart 2 describes the children participating in the study, according to the groups, including the phonological processes and target sounds addressed in PROCICLOS-A.

**Chart 2:** Descriptive data of the children in the study at the initial assessment

Groups	Participants	Age	Sex	Target Phonological Processes	Target Sounds
C1	Child 1	5:11	Male	LS, VF	/ɾ/, /ʎ/, /k/, /g/
	Child 2	6:8	Female	FD, PD	/v/, /z/, /b/, /d/
	Child 3	7:7	Male	VF, PF	/k/, /g/, /ʃ/, /ʒ/
	Child 4	5:6	Male	VF, FD	/k/, /g/, /v/, /z/
C2	Child 5	6:0	Female	PF, CCS	/ʃ/, /ʒ/, Enc _R
	Child 6	5:3	Male	PF, FD	/ʃ/, /ʒ/, /v/, /z/
	Child 7	5:3	Male	PF, FD	/ʃ/, /ʒ/, /v/, /z/
	Child 8	6:5	Male	CCS	Enc /l/ Enc/ɾ/

**Caption:** C1: group once a week; C2: group twice a week; VF: Velar Fronting; PF: Palatal Fronting; LS: Liquid Simplification; CCS: Consonant Cluster Simplification; PD: Plosive Devoicing; FD: Fricative Devoicing

### Efficacy of PROCICLOS-A

PCC, PCC-R, PDI, and NPP were compared at the three assessment times to verify the efficacy of PROCICLOS-A in each group. The results presented in Tables 1 and 2 show a significant difference for all measures, except for NPP-Naming in C1 and C2 and PCC-Imitation in C2. It is noteworthy that performance improved in the assessments after the intervention.

**Table 1:** Descriptive values ​​and comparative analysis of moments according to group and task

Group	Measure	Moment	n	Naming						
				Mean	SD	Median	Min	Max	p	ES
C1	NPP	A1	4	4.25	0.96	4.50	3.00	5.00	0.093	0.544^†^
		A2	4	2.75	2.22	3.00	0.00	5.00		
		A3	4	2.50	1.73	2.50	1.00	4.00		
	PCC	A1	4	67.75	13.72	66.10	54.40	84.40	0.009*	0.925^†^
		A2	4	83.30	12.16	83.30	70.00	96.60		
		A3	4	84.43	12.76	85.00	70.00	97.70		
	PCC-R	A1	4	68.88	12.83	66.65	57.80	84.40	0.009*	0.925^†^
		A2	4	83.78	11.84	84.25	70.00	96.60		
		A3	4	84.98	12.42	86.10	70.00	97.70		
	PDI	A1	4	0.82	0.34	0.89	0.41	1.10	0.005*	0.925^†^
		A2	4	0.50	0.39	0.51	0.08	0.88		
		A3	4	0.42	0.37	0.37	0.06	0.88		
C2	NPP	A1	4	4.00	2.31	4.00	2.00	6.00	0.074	0.465^†^
		A2	4	3.25	3.20	3.50	0.00	6.00		
		A3	4	2.00	1.83	2.00	0.00	4.00		
	PCC	A1	4	66.68	12.31	62.25	57.80	84.40	0.042*	0.720^†^
		A2	4	75.80	15.30	73.30	62.20	94.40		
		A3	4	79.95	12.31	75.50	71.10	97.70		
	PCC-R	A1	4	71.68	15.40	71.65	57.80	85.60	0.005*	1.000^†^
		A2	4	79.73	18.35	80.00	62.20	96.70		
		A3	4	86.88	13.42	88.25	72.20	98.80		
	PDI	A1	4	0.76	0.40	0.77	0.38	1.12	0.005*	1.000^†^
		A2	4	0.53	0.49	0.53	0.09	1.00		
		A3	4	0.36	0.36	0.32	0.05	0.74		
Total	NPP	A1	8	4.12	1.64	4.50	2.00	6.00	0.002*	0.484^†^
		A2	8	3.00	2.56	3.00	0.00	6.00		
		A3	8	2.25	1.67	2.00	0.00	4.00		
	PCC	A1	8	67.21	12.08	62.25	54.40	84.40	< 0.001*	0.813^†^
		A2	8	79.55	13.41	79.40	62.20	96.60		
		A3	8	82.19	11.85	78.30	70.00	97.70		
	PCC-R	A1	8	70.27	13.21	66.65	57.80	85.60	< 0.001*	0.958^†^
		A2	8	81.75	14.46	84.25	62.20	96.70		
		A3	8	85.93	12.01	86.10	70.00	98.80		
	PDI	A1	8	0.79	0.34	0.89	0.38	1.12	< 0.001*	0.958^†^
		A2	8	0.51	0.41	0.51	0.08	1.00		
		A3	8	0.39	0.34	0.37	0.05	0.88		
Group	Measure	Moment	n	Imitation						
				Mean	SD	Median	Min	Max	p	ES
C1	NPP	A1	4	4.00	1.41	4.50	2.00	5.00	0.037*
		A2	4	2.25	2.06	2.50	0.00	4.00	
		A3	4	2.50	1.73	2.50	1.00	4.00	
	PCC	A1	4	67.50	5.98	67.75	60.70	73.80	0.009*	0.925^†^
		A2	4	82.63	12.18	82.20	68.90	97.20		
		A3	4	86.90	10.62	87.80	74.80	97.20		
	PCC-R	A1	4	68.68	5.81	70.10	60.70	73.80	0.009*	0.925^†^
		A2	4	82.63	12.18	82.20	68.90	97.20		
		A3	4	87.35	9.96	87.80	76.60	97.20		
	PDI	A1	4	0.87	0.15	0.85	0.72	1.07	0.009*	0.925^†^
		A2	4	0.49	0.35	0.52	0.07	0.85		
		A3	4	0.36	0.28	0.37	0.07	0.61		
C2	NPP	A1	4	4.00	2.45	3.50	2.00	7.00	0.037*	0.786^†^
		A2	4	1.75	2.06	1.50	0.00	4.00		
		A3	4	1.75	2.06	1.50	0.00	4.00		
	PCC	A1	4	66.88	10.02	63.55	58.90	81.50	0.069	0.625^†^
		A2	4	83.70	11.46	83.20	71.00	97.40		
		A3	4	82.93	9.08	79.45	76.60	96.20		
	PCC-R	A1	4	71.78	12.86	72.05	58.90	84.10	0.042*	0.720^†^
		A2	4	86.43	13.41	88.30	71.00	98.10		
		A3	4	88.30	10.36	88.75	77.60	98.10		
	PDI	A1	4	0.78	0.35	0.78	0.44	1.13	0.042*	0.720^†^
		A2	4	0.37	0.36	0.32	0.05	0.79		
		A3	4	0.31	0.30	0.29	0.05	0.62		
Total	NPP	A1	8	4.00	1.85	4.50	2.00	7.00	< 0.001*	0.841^†^
		A2	8	2.00	1.93	2.00	0.00	4.00		
		A3	8	2.13	1.81	2.00	0.00	4.00		
	PCC	A1	8	67.19	7.65	64.50	58.90	81.50	< 0.001*	0.715^†^
		A2	8	83.16	10.96	82.70	68.90	97.40		
		A3	8	84.91	9.39	81.30	74.80	97.20		
	PCC-R	A1	8	70.22	9.39	70.10	58.90	84.10	< 0.001*	0.813^†^
		A2	8	84.53	12.04	83.15	68.90	98.10		
		A3	8	87.83	9.42	87.80	76.60	98.10		
	PDI	A1	8	0.83	0.25	0.85	0.44	1.13	< 0.001*	0.813†
		A2	8	0.43	0.34	0.46	0.05	0.85		
		A3	8	0.33	0.27	0.33	0.05	0.62		

* statistically significant value at 5% (p ≤ 0.05);

† Effect size greater than or equal to moderate/medium.

**Caption:** SD: Standard deviation; Min.: Minimum; Max.: Maximum; ES: Effect size

**Table 2:** Descriptive values ​​and comparative analysis of ABFW Phonology measures between C1 and C2, according to the assessment time

Moment	Measure	Group	n	Naming						
				Mean	SD	Median	Min	Max	p	ES
A1	NPP	C1	4	4.25	0.96	4.50	3.00	5.00	> 0.999	0.000
		C2	4	4.00	2.31	4.00	2.00	6.00		
	PCC	C1	4	67.75	13.72	66.10	54.40	84.40	> 0.999	0.000
		C2	4	66.68	12.31	62.25	57.80	84.40		
	PCC-R	C1	4	68.88	12.83	66.65	57.80	84.40	0.829	0.156
		C2	4	71.68	15.40	71.65	57.80	85.60		
	PDI	C1	4	0.82	0.34	0.89	0.41	10.10	0.971	0.051
		C2	4	0.76	0.40	0.77	0.38	10.12		
A2	NPP	C1	4	2.75	2.22	3.00	0.00	5.00	0.743	0.155
		C2	4	3.25	3.20	3.50	0.00	6.00		
	PCC	C1	4	83.30	12.16	83.30	70.00	96.60	0.486	0.306^†^
		C2	4	75.80	15.30	73.30	62.20	94.40		
	PCC-R	C1	4	83.78	11.84	84.25	70.00	96.60	0.886	0.102
		C2	4	79.73	18.35	80.00	62.20	96.70		
	PDI	C1	4	0.50	0.39	0.51	0.08	0.88	0.686	0.204
		C2	4	0.53	0.49	0.53	0.09	1.00		
A3	NPP	C1	4	2.50	1.73	2.50	1.00	4.00	0.743	0.215
		C2	4	2.00	1.83	2.00	0.00	4.00		
	PCC	C1	4	84.43	12.76	85.00	70.00	97.70	0.971	0.051
		C2	4	79.95	12.31	75.50	71.10	97.70		
	PCC-R	C1	4	84.98	12.42	86.10	70.00	97.70	0.743	0.154
		C2	4	86.88	13.42	88.25	72.20	98.80		
	PDI	C1	4	0.42	0.37	0.37	0.06	0.88	0.600	0.205
		C2	4	0.36	0.36	0.32	0.05	0.74		
Moment	Measure	Group	n	Imitation						
				Mean	SD	Median	Min	Max	p	ES
A1	NPP	C1	4	4.00	1.41	4.50	2.00	5.00	> 0.999	0.000
		C2	4	4.00	2.45	3.50	2.00	7.00		
	PCC	C1	4	67.50	5.98	67.75	60.70	73.80	0.743	0.154
		C2	4	66.88	10.02	63.55	58.90	81.50		
	PCC-R	C1	4	68.68	5.81	70.10	60.70	73.80	0.886	0.102
		C2	4	71.78	12.86	72.05	58.90	84.10		
	PDI	C1	4	0.87	0.15	0.85	0.72	1.07	0.886	0.102
		C2	4	0.78	0.35	0.78	0.44	1.13		
A2	NPP	C1	4	2.25	2.06	2.50	0.00	4.00	0.743	0.215
		C2	4	1.75	2.06	1.50	0.00	4.00		
	PCC	C1	4	82.63	12.18	82.20	68.90	97.20	0.686	0.204
		C2	4	83.70	11.46	83.20	71.00	97.40		
	PCC-R	C1	4	82.63	12.18	82.20	68.90	97.20	0.543	0.257
		C2	4	86.43	13.41	88.30	71.00	98.10		
	PDI	C1	4	0.49	0.35	0.52	0.07	0.85	0.686	0.204
		C2	4	0.37	0.36	0.32	0.05	0.79		
A3	NPP	C1	4	2.50	1.73	2.50	1.00	4.00	0.400	0.318†
		C2	4	1.75	2.06	1.50	0.00	4.00		
	PCC	C1	4	86.90	10.62	87.80	74.80	97.20	0.743	0.154
		C2	4	82.93	9.08	79.45	76.60	96.20		
	PCC-R	C1	4	87.35	9.96	87.80	76.60	97.20	0.743	0.154
		C2	4	88.30	10.36	88.75	77.60	98.10		
	PDI	C1	4	0.36	0.28	0.37	0.07	0.61	0.743	0.154
		C2	4	0.31	0.30	0.29	0.05	0.62		

Wilcoxon signed-rank test

† Effect size greater than or equal to moderate/medium

**Caption:** SD: Standard deviation; Min: Minimum; Max: Maximum; ES: Effect size

The ES was also analyzed, finding results that varied between moderate and large for all measures at different evaluation moments in both C1 and C2. This indicates better performance of the children after applying PROCICLOS-A.

### Analysis of the Influence of Dose and Session Frequency

The performance of C1 and C2 in the three evaluation moments was used to analyze the influence of the frequency dose of the sessions. Table 2 shows no significant difference between C1 and C2 in relation to NPP, PCC, PCC-R, or PDI in the ABFW Phonology tests (naming and imitation) in the three evaluation moments. However, the ES shows moderate to large values in the PCC-Naming measures for C1 in A2, and for NPP-Imitation for C2 in A3. The analyses indicate that C1 and C2 had similar performances in A1, and, after PROCICLOS-A, a large ES was observed for C1, and a moderate ES for C2.

### Performance in PROCICLOS-A sessions

The scoring system was used for each of the 12 sessions and for the activities to analyze the children’s mean performance in the sessions. Table 3 presents the PCC, PCC-R, and PDI in the initial assessment per child, and the mean performance in the 12 sessions. The latter was obtained through each session’s score, then calculating the performance in percentage and the mean performance in the 12 PROCICLOS-A sessions per child.

**Table 3:** Values ​​of ABFW phonology measures in A1 and children‘s mean performance in the 12 PROCICLOS-A sessions.

Group	Participants	PDI		PCC (%)		PCC-R (%)		Mean performance in the 12 sessions
		A1		A1		A1		
		N	I	N	I	N	I	
C1	Child 1	0.68	0.82	73.3	71.0	74.4	72.0	73%
	Child 2	1.09	0.87	54.4	64.5	57.8	68.2	86%
	Child 3	0.41	0.72	84.4	73.8	84.4	73.8	85%
	Child 4	1.1	1.07	58.9	60.7	58.9	60.7	68%
C2	Child 5	0.38	0.44	65.6	64.5	85.6	84.1	72%
	Child 6	1.09	1.13	58.9	58.9	58.9	58.9	83%
	Child 7	1.12	1.03	57.8	62.6	57.8	62.6	68%
	Child 8	0.44	0.52	84.4	81.5	84.4	81.5	64%

**Caption:** A1: initial assessment. N: naming; I: imitation

**Figure 2:**
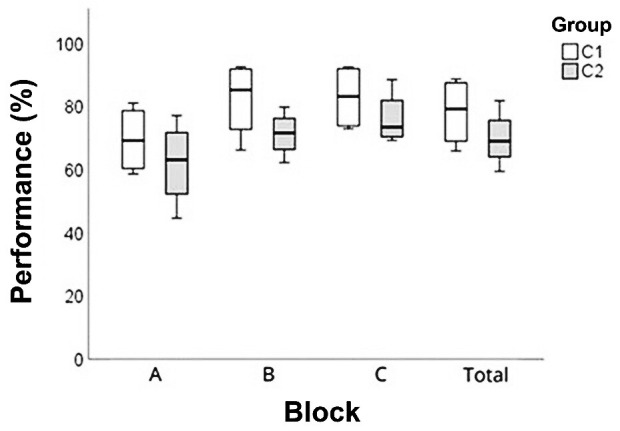
Measures of central tendency and dispersion of performance in the sessions

Figure 2 shows the measures of central tendency and dispersion of the performance of the sessions grouped into blocks of sessions: A (sessions 1, 4, 7, and 10), B (sessions 2, 5, 8, and 11), C (sessions 3, 6, 9, and 12) and the total number of sessions, through descriptive data analysis. The children obtained a lower performance in the sessions of block A, which correspond to the presentation of a new target sound. Observing the total performance, C1 had a higher average performance (78%) than C2 (72%).

**Figure 3:**
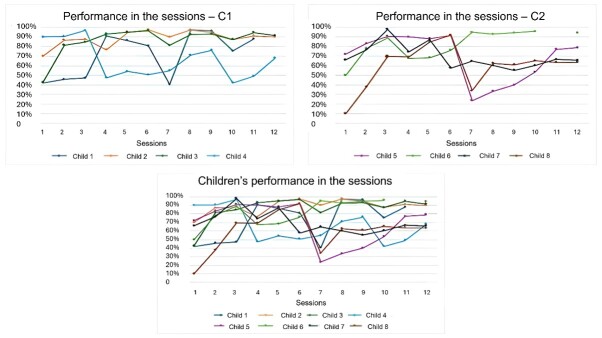
Each child’s mean performance the 12 PROCICLOS-A sessions

Each child’s mean performance for each of the 12 sessions is presented in Figure 3. Children performed worse in the sessions of block A and improved in performance in the sessions of blocks B and C.

Spearman's correlation was analyzed between the mean performance in each of the blocks of sessions A, B, and C and each of the measures of the ABFW phonology tests obtained in assessments A1, A2, and A3 (Table 4). No statistically significant correlations were observed for any of the analyses.

**Table 4:** Correlation between performance in blocks of sessions with ABFW phonology measures according to the time of assessment.

	Session	Task		C1 (n = 4)					
				A1	A2	A3	A2-A1	A3-A1	A3-A2
NPP	A	N	Coef.	-0.105	0.200	0.000	0.200	0.105	-0.447^†^
			p	0.895	0.800	> 0.999	0.800	0.895	0.553
		I	Coef.	-0.105	-0.105	0.000	0.105	0.258	NC
			p	0.895	0.895	> 0.999	0.895	0.742	NC
	B	N	Coef.	-0.105	0.200	0.000	0.200	0.105	-0.447^†^
			p	0.895	0.800	> 0.999	0.800	0.895	0.553
		I	Coef.	-0.105	-0.105	0.000	0.105	0.258	NC
			p	0.895	0.895	> 0.999	0.895	0.742	NC
	C	N	Coef.	-0.105	0.200	0.000	0.200	0.105	-0.447^†^
			p	0.895	0.800	> 0.999	0.800	0.895	0.553
		I	Coef.	-0.105	-0.105	0.000	0.105	0.258	NC
			p	0.895	0.895	> 0.999	0.895	0.742	NC
	Total	N	Coef.	-0.105	0.200	0.000	0.200	0.105	-0.447^†^
			p	0.895	0.800	> 0.999	0.800	0.895	0.553
		I	Coef.	-0.105	-0.105	0.000	0.105	0.258	NC
			p	0.895	0.895	> 0.999	0.895	0.742	NC
PCC	A	N	Coef.	-0.200	0.400^†^	0.400^†^	0.800^†^	0.800^†^	0.400^†^
			p	0.800	0.600	0.600	0.200	0.200	0.600
		I	Coef.	0.400^†^	-0.200	-0.200	-0.400^†^	-0.200	0.000
			p	0.600	0.800	0.800	0.600	0.800	> 0.999
	B	N	Coef.	-0.200	0.400^†^	0.400^†^	0.800^†^	0.800^†^	0.400^†^
			p	0.800	0.600	0.600	0.200	0.200	0.600
		I	Coef.	0.400^†^	-0.200	-0.200	-0.400^†^	-0.200	0.000
			p	0.600	0.800	0.800	0.600	0.800	> 0.999
	C	N	Coef.	-0.200	0.400^†^	0.400^†^	0.800^†^	0.800^†^	0.400^†^
			p	0.800	0.600	0.600	0.200	0.200	0.600
		I	Coef.	0.400^†^	-0.200	-0.200	-0.400^†^	-0.200	0.000
			p	0.600	0.800	0.800	0.600	0.800	> 0.999
	Total	N	Coef.	-0.200	0.400^†^	0.400^†^	0.800^†^	0.800^†^	0.400^†^
			p	0.800	0.600	0.600	0.200	0.200	0.600
		I	Coef.	0.400^†^	-0.200	-0.200	-0.400^†^	-0.200	0.000
			p	0.600	0.800	0.800	0.600	0.800	> 0.999
PCC-R	A	N	Coef.	-0.200	0.400^†^	0.400^†^	0.800^†^	0.800^†^	0.400^†^
			p	0.800	0.600	0.600	0.200	0.200	0.600
		I	Coef.	0.400^†^	-0.200	-0.200	-0.400^†^	-0.200	0.400^†^
			p	0.600	0.800	0.800	0.600	0.800	0.600
	B	N	Coef.	-0.200	0.400^†^	0.400^†^	0.800^†^	0.800^†^	0.400^†^
			p	0.800	0.600	0.600	0.200	0.200	0.600
		I	Coef.	0.400^†^	-0.200	-0.200	-0.400^†^	-0.200	0.400^†^
			p	0.600	0.800	0.800	0.600	0.800	0.600
	C	N	Coef.	-0.200	0.400^†^	0.400^†^	0.800^†^	0.800^†^	0.400^†^
			p	0.800	0.600	0.600	0.200	0.200	0.600
		I	Coef.	0.400^†^	-0.200	-0.200	-0.400^†^	-0.200	0.400^†^
			p	0.600	0.800	0.800	0.600	0.800	0.600
	Total	N	Coef.	-0.200	0.400^†^	0.400^†^	0.800^†^	0.800^†^	0.400^†^
			p	0.800	0.600	0.600	0.200	0.200	0.600
		I	Coef.	0.400^†^	-0.200	-0.200	-0.400^†^	-0.200	0.400^†^
			p	0.600	0.800	0.800	0.600	0.800	0.600
PDI	A	N	Coef.	-0.400^†^	-0.400^†^	-0.400^†^	-0.400^†^	-0.800^†^	-0.800^†^
			p	0.600	0.600	0.600	0.600	0.200	0.200
		I	Coef.	-0.400^†^	0.200	0.200	0.200	0.400^†^	-0.400^†^
			p	0.600	0.800	0.800	0.800	0.600	0.600
	B	N	Coef.	-0.400^†^	-0.400^†^	-0.400^†^	-0.400^†^	-0.800^†^	-0.800^†^
			p	0.600	0.600	0.600	0.600	0.200	0.200
		I	Coef.	-0.400^†^	0.200	0.200	0.200	0.400^†^	-0.400^†^
			p	0.600	0.800	0.800	0.800	0.600	0.600
	C	N	Coef.	-0.400^†^	-0.400^†^	-0.400^†^	-0.400^†^	-0.800^†^	-0.800^†^
			p	0.600	0.600	0.600	0.600	0.200	0.200
		I	Coef.	-0.400^†^	0.200	0.200	0.200	0.400^†^	-0.400^†^
			p	0.600	0.800	0.800	0.800	0.600	0.600
	Total	N	Coef.	-0.400^†^	-0.400^†^	-0.400^†^	-0.400^†^	-0.800^†^	-0.800^†^
			P	0.600	0.600	0.600	0.600	0.200	0.200
		I	Coef.	-0.400^†^	0.200	0.200	0.200	0.400^†^	-0.400^†^
			P	0.600	0.800	0.800	0.800	0.600	0.600
	Session	Task		C2 (n = 4)					
				A1	A2	A3	A2-A1	A3-A1	A3-A2
NPP	A	N	Coef.	0.894^†^	0.949^†^	0.600^†^	0.949^†^	-0.949^†^	-1000^†^
			p	0.106	0.051	0.400	0.051	0.051	--
		I	Coef.	0.949^†^	0.949^†^	0.738^†^	-0.775^†^	-0.316^†^	-0.775^†^
			p	0.051	0.051	0.262	0.225	0.684	0.225
	B	N	Coef.	0.894^†^	0.949^†^	0.600^†^	0.949^†^	-0.949^†^	-1000^†^
			p	0.106	0.051	0.400	0.051	0.051	--
		I	Coef.	0.949^†^	0.949^†^	0.738^†^	-0.775^†^	-0.316^†^	-0.775^†^
			p	0.051	0.051	0.262	0.225	0.684	0.225
	C	N	Coef.	0.000	0.105	-0.400	0.105	-0.632^†^	-0.400^†^
			p	> 0.999	0.895	0.600	0.895	0.368	0.600
		I	Coef.	0.316	0.316	0.316	-0.775^†^	-0.949^†^	-0.775^†^
			p	0.684	0.684	0.684	0.225	0.051	0.225
	Total	N	Coef.	0.894^†^	0.949^†^	0.600^†^	0.949^†^	-0.949^†^	-1000^†^
			p	0.106	0.051	0.400	0.051	0.051	--
		I	Coef.	0.949^†^	0.949^†^	0.738^†^	-0.775^†^	-0.316^†^	-0.775^†^
			p	0.051	0.051	0.262	0.225	0.684	0.225
PCC	A	N	Coef.	-0.800^†^	-0.800^†^	-0.400^†^	-0.600^†^	0.600^†^	0.600^†^
			p	0.200	0.200	0.600	0.400	0.400	0.400
		I	Coef.	-1.000^†^	-1.000^†^	-0.400^†^	-0.800^†^	0.738^†^	0.949^†^
			p	--	--	0.600	0.200	0.262	0.051
	B	N	Coef.	-0.800^†^	-0.800^†^	-0.400^†^	-0.600^†^	0.600^†^	0.600^†^
			p	0.200	0.200	0.600	0.400	0.400	0.400
		I	Coef.	-1.000^†^	-1.000^†^	-0.400^†^	-0.800^†^	0.738^†^	0.949^†^
			p	--	--	0.600	0.200	0.262	0.051
	C	N	Coef.	0.200	0.200	-0.600^†^	0.400^†^	-0.400^†^	-0.400^†^
			p	0.800	0.800	0.400	0.600	0.600	0.600
		I	Coef.	-0.400^†^	-0.400^†^	-0.600^†^	-0.200	-0.105	0.316^†^
			p	0.600	0.600	0.400	0.800	0.895	0.684
	Total	N	Coef.	-0.800^†^	-0.800^†^	-0.400^†^	-0.600^†^	0.600^†^	0.600^†^
			p	0.200	0.200	0.600	0.400	0.400	0.400
		I	Coef.	-1.000^†^	-1.000^†^	-0.400^†^	-0.800^†^	0.738^†^	0.949^†^
			p	--	--	0.600	0.200	0.262	0.051
PCC-R	A	N	Coef.	-0.600^†^	-0.600^†^	-0.800^†^	-0.600^†^	0.600^†^	0.600^†^
			p	0.400	0.400	0.200	0.400	0.400	0.400
		I	Coef.	-0.800^†^	-1.000^†^	-0.800^†^	-0.400^†^	0.738^†^	1.000^†^
			p	0.200	--	0.200	0.600	0.262	--
	B	N	Coef.	-0.600^†^	-0.600^†^	-0.800^†^	-0.600^†^	0.600^†^	0.600^†^
			p	0.400	0.400	0.200	0.400	0.400	0.400
		I	Coef.	-0.800^†^	-1.000^†^	-0.800^†^	-0.400^†^	0.738^†^	1.000^†^
			p	0.200	--	0.200	0.600	0.262	--
	C	N	Coef.	0.400^†^	0.400^†^	-0.200	0.400^†^	-0.400^†^	-0.400^†^
			p	0.600	0.600	0.800	0.600	0.600	0.600
		I	Coef.	-0.200	-0.400^†^	-0.200	-1.000^†^	-0.105	0.400^†^
			p	0.800	0.600	0.800	--	0.895	0.600
	Total	N	Coef.	-0.600^†^	-0.600^†^	-0.800^†^	-0.600^†^	0.600^†^	0.600^†^
			p	0.400	0.400	0.200	0.400	0.400	0.400
		I	Coef.	-0.800^†^	-1.000^†^	-0.800^†^	-0.400^†^	0.738^†^	1.000^†^
			p	0.200	--	0.200	0.600	0.262	--
PDI	A	N	Coef.	0.600^†^	0.600^†^	0.949^†^	0.800^†^	0.000	-0.600^†^
			p	0.400	0.400	0.051	0.200	> 0.999	0.400
		I	Coef.	0.800^†^	1.000^†^	0.800^†^	0.800^†^	-0.600^†^	-1.000^†^
			p	0.200	--	0.200	0.200	0.400	--
	B	N	Coef.	0.600^†^	0.600^†^	0.949^†^	0.800^†^	0.000	-0.600^†^
			p	0.400	0.400	0.051	0.200	> 0.999	0.400
		I	Coef.	0.800^†^	1.000^†^	0.800^†^	0.800^†^	-0.600^†^	-1.000^†^
			p	0.200	--	0.200	0.200	0.400	--
	C	N	Coef.	-0.400^†^	0.400^†^	0.316^†^	-0.200	0.800^†^	0.400^†^
			p	0.600	0.600	0.684	0.800	0.200	0.600
		I	Coef.	0.200	0.400^†^	0.200	0.800^†^	0.400^†^	-0.400^†^
			p	0.800	0.600	0.800	0.200	0.600	0.600
	Total	N	Coef.	0.600^†^	0.600^†^	0.949^†^	0.800^†^	0.000	-0.600^†^
			P	0.400	0.400	0.051	0.200	1.000	0.400
		I	Coef.	0.800^†^	1.000^†^	0.800^†^	0.800^†^	-0.600^†^	-1.000^†^
			P	0.200	--	0.200	0.200	0.400	--
	Session	Task		Total (n = 8)					
				A1	A2	A3	A2-A1	A3-A1	A3-A2
NPP	A	N	Coef.	0.558^†^	0.518^†^	0.350^†^	0.469^†^	-0.315^†^	-0.551^†^
			p	0.151	0.188	0.395	0.241	0.447	0.157
		I	Coef.	0.501^†^	0.451^†^	0.469^†^	-0.077	0.089	-0.412^†^
			p	0.206	0.263	0.241	0.856	0.833	0.310
	B	N	Coef.	0.364^†^	0.289	0.200	0.210	-0.315^†^	-0.350^†^
			p	0.300	0.487	0.634	0.618	0.447	0.395
		I	Coef.	0.300^†^	0.300^†^	0.346^†^	-0.077	0.115	-0.247
			p	0.470	0.470	0.401	0856	0.786	0.555
	C	N	Coef.	-0.036	-0.048	-0.100	0.025	-0.101	-0.050
			p	0.932	0.910	0.814	0.954	0.812	0.906
		I	Coef.	0.050	0.150	0.025	0.077	0.000	-0.247
			p	0.906	0.723	0.954	0.856	> 0.999	0.555
	Total	N	Coef.	0.364^†^	0.289	0.200	0.210	-0.315^†^	-0.350^†^
			p	0.376	0.487	0.634	0.618	0.447	0.395
		I	Coef.	0.300^†^	0.300^†^	0.346^†^	-0.077	0.115	-0.247
			p	0.470	0.470	0.401	0.856	0.786	0.555
PCC	A	N	Coef.	-0.506^†^	-0.262	0.012	0.190	0.659^†^	0.252
			p	0.201	0.531	0.978	0.651	0.076	0.548
		I	Coef.	-0.299	-0.619^†^	-0.395^†^	-0.554^†^	-0.072	0.415^†^
			p	0.471	0.102	0.333	0.154	0.866	0.307
	B	N	Coef.	-0.313^†^	0.000	0.144	0.405^†^	0.599^†^	0.084
			p	0.450	> 0.999	0.734	0.320	0.117	0.844
		I	Coef.	-0.084	-0.476^†^	-0.240	-0.386^†^	0.072	0.390^†^
			p	0.844	0.233	0.568	0.346	0.866	0.339
	C	N	Coef.	-0.096	0.262	-0.060	0.619^†^	0.132	-0.359^†^
			p	0.820	0.531	0.888	0.102	0.756	0.382
		I	Coef.	0.012	-0.333^†^	-0.335^†^	-0.096	-0.108	0.171
			p	0.978	0.420	0.417	0.820	0.799	0.686
	Total	N	Coef.	-0.313^†^	0.000	0.144	0.405^†^	0.599^†^	0.084
			p	0.450	> 0.999	0.734	0.320	0.117	0.844
		I	Coef.	-0.084	-0.476^†^	-0.240	-0.386^†^	0.072	0.390^†^
			p	0.844	0.233	0.568	0.346	0.866	0.339
PCC-R	A	N	Coef.	-0.521^†^	-0.310^†^	-0.228	0.262	0.683^†^	0.071
			p	0.185	0.456	0.588	0.531	0.062	0.867
		I	Coef.	-0.405^†^	-0.659^†^	-0.563^†^	-0.357^†^	-0.072	0.515^†^
			p	0.320	0.076	0.146	-0.286	0.072	0.479
	B	N	Coef.	-0.364^†^	-0.095	-0.072	0.500^†^	0.623^†^	-0.119
			p	0.376	0.823	0.866	0.207	0.099	0.779
		I	Coef.	-0.238	-0.527^†^	-0.407^†^	-0.286	0.072	0.479^†^
			p	0.570	0.180	0.317	0.493	0.866	0.230
	C	N	Coef.	0.036	0.310^†^	0.156	0.667^†^	0.180	-0.452^†^
			p	0.932	0.456	0.713	0.071	0.670	0.260
		I	Coef.	0.024	-0.359^†^	-0.168	-0.476^†^	-0.108	0.323^†^
			p	0.955	0.382	0.691	0.233	0.799	0.435
	Total	N	Coef.	-0.364^†^	-0.095	-0.072	0.500^†^	0.623^†^	-0.119
			p	0.376	0.823	0.866	0.207	0.099	0.779
		I	Coef.	-0.238	-0.527^†^	-0.407^†^	-0.286	0.072	0.479^†^
			p	0.570	0.180	0.317	0.493	0.866	0.230
PDI	A	N	Coef.	0.204	0.238	0.311^†^	-0.095	-0.476^†^	-0.476^†^
			p	0.629	0.570	0.453	0.823	0.233	0.233
		I	Coef.	0.405^†^	0.619^†^	0.563^†^	0.443^†^	-0.048	-0.515^†^
			p	0.320	0.102	0.146	0.272	0.911	0.192
	B	N	Coef.	0.024	0.000	0.168	-0.357^†^	-0.429^†^	-0.286
			p	0.955	> 0.999	0.691	0.385	0.289	0.493
		I	Coef.	0.238	0.476^†^	0.407^†^	0.299	-0.143	-0.479†
			p	0.570	0.233	0.317	0.471	0.736	0.230
	C	N	Coef.	-0.359^†^	-0.357^†^	-0.060	-0.476^†^	-0.024	0.095
			p	0.382	0.385	0.888	0.233	0.955	0.823
		I	Coef.	-0.024	0.333^†^	0.204	0.419^†^	0.214	-0.323^†^
			p	0.955	0.420	0.629	0.301	0.610	0.435
	Total	N	Coef.	0.024	0.000	0.168	-0.357^†^	-0.429^†^	-0.286
			P	0.955	> 0.999	0.691	0.385	0.289	0.493
		I	Coef.	0.238	0.476^†^	0.407^†^	0.299	-0.143	-0.479^†^
			P	0.570	0.233	0.317	0.471	0.736	0.230

Spearman's correlation test

† average/moderate or greater effect

**Caption:** Coef.: correlation coefficient; A: Average of sessions 1, 4, 7, and 10; B: Average of sessions 2, 5, 8, and 11; C: Average of sessions 3, 6, 9, and 12; N: Naming; I: Imitation

## DISCUSSION

Some studies have discussed the efficacy of SSD intervention approaches^(9,11,40)^. Specifically, interventions in SSD of unknown cause and of the phonological type are the most studied^(13,17)^, among which is the Cycles approach^(23)^. This study investigated the efficacy of PROCICLOS-A in children with SSD and phonological impairment, comparing the children's performance at three different times: before the program was implemented, immediately after the program was implemented, and 6 weeks after the end of the program.

### Efficacy of PROCICLOS-A

The study results indicate evidence of the efficacy of PROCICLOS-A, as better results were observed for each of the children, regardless of the frequency of the sessions, in the post-intervention evaluations (A2 and A3). The inferential analysis considering NPP, PCC, PCC-R, and PDI in the three evaluation moments (A1, A2, and A3) found a significant difference for all, except for NPP in the Naming test and PCC in the Imitation test, both for C1 and C2. Regarding NPP, it is worth noting that many times a phonological process is still present in the speech of a child with SSD after the first intervention cycle, but its occurrence decreases^(24)^. Regarding PCC, one factor that can interfere is that sound distortions are considered errors. The ES observed in the various analyses reinforces the evidence of the efficacy of PROCICLOS-A, since the values ​​were between moderate and large for all measures for C1 and C2. This indicates better performance by children after the application of PROCICLOS-A, regardless of the frequency to which they were subjected.

PROCICLOS-A is an intervention program with a cycle of 12 sessions, in which two phonological processes and four target sounds are worked on, preferably those that are stimulable, considering the proposal of the Hodson and Paden Cycle approach^(23)^. Thus, the child is exposed to new sounds before the previous target sound is necessarily mastered^(24)^, stimulating the child with SSD to the tendency to generalize and eliminate error patterns gradually.

Another important highlight of the efficacy of PROCICLOS-A is the six activities developed in all sessions, which work on different skills, including auditory stimulation and articulation of the target sound, and the use of minimal pairs and phonological awareness. Farquharson^(41)^ mentions that children with SSD need approaches that encompass different skills to overcome the difficulties of each case, and this type of approach can have better results than those focused on a single skill.

Stimulating different skills in the intervention for SSD of unknown cause was also highlighted by Brosseau-Lapré and Roepke^(5)^, in an approach that involves auditory perception and speech production, effective for children with SSD. The authors recommend that interventions ensure that children develop good perceptual knowledge of the target sound, provide production practice with sufficient intensity, and incorporate metaphonological activities during speech therapy to promote accuracy of speech production in the therapeutic environment, generalization to spontaneous speech, and establish the foundation for reading skills.

### Analysis of the Influence of Dose and Session Frequency

One of the objectives of the present study was to verify the influence of the weekly frequency of sessions on the efficacy of PROCICLOS-A. Exploring this issue was motivated by a common doubt about what is best for children with SSD. However, the influence of the dose and frequency of sessions is still a topic discussed in the literature and deserves the attention of researchers to identify which components of intervention intensity most influence the efficacy of intervention approaches^(9,20,22,40,42)^.

Some factors may interfere with the performance of a child with SSD during the intervention, regardless of the approach. Among these is the cumulative intensity of the intervention, proposed by Warren et al.^(43)^, which can provide a general indicator of the total intensity of an intervention. Three components are important to calculate this index: the first is the dose of moments of stimulation of the intervention target; the second is the frequency of the dose of the intervention sessions in a period, such as per week; and the total duration of the intervention, such as the number of weeks. The cumulative intensity of intervention has been indicated as an important aspect for analyzing intervention approaches^(13)^. In addition to the three components of the cumulative intensity of the intervention, Warren et al.^(43)^ indicate that the form of the dose is an important element of the intervention. This refers to the typical task or activity applied to stimulate the intervention target, and the form of the dose facilitates the identification of the dose.

The variation of each of the components of cumulative intensity can be studied, which would allow identifying which of them would provide better intervention results. In PROCICLOS-A, the four components were structured with the form of the dose established through the activities and strategies for each session, the dose of stimuli per session around 100 for 50 minutes, the frequency of the sessions once or twice a week for C1 or C2, and the total duration of the cycle was 12 weeks for C1 and 6 weeks for C2. Thus, in this study, the controlled variables were the form of the dose, the dose and cumulative intensity of the intervention, and the independent variables were the dose frequency and the total duration of the intervention. The cumulative intensity of the intervention^(43)^ is calculated considering the dose x frequency of the sessions x total duration of the intervention, which results in 1200 for both C1 and C2. Considering this concept of intervention intensity, this was the same for both groups, since for C1 the intervention lasted 12 weeks and for C2 it lasted 6 weeks. The study results suggest that the total duration of the intervention, in number of hours, was more important for the improvement of the children, making the frequency of once or twice a week a variable with less impact.

### Performance in PROCICLOS-A sessions

In PROCICLOS-A, the form of the dose – i.e., the definition of the activities applied in the sessions – was previously outlined to reach the skills that interfere with speech^(5,11,23,42)^. In addition to the activities, all stimuli were selected according to the target sounds involved in the error patterns or phonological processes most observed in children with SSD who speak Brazilian Portuguese.

As indicated in the method, a scoring system for the activities was created to verify the performance of the children in the study, with the total expected for each target sound calculated based on the stimuli present in the sessions. This enables the observation of the percentage of correct answers obtained in the sessions and specifically in each of the activities, indicating the skills in which there was greater or lesser difficulty for children with SSD.

The results of the analysis of performance during the intervention sessions showed that children with SSD made good use of the activities that involved auditory perception, phonological awareness, minimal pairs, and articulatory training, which are skills identified as deficient in children with SSD and phonological impairment^(5,11,44)^.

The children’s performance per session was not statistically significantly correlated with the improvement observed in the measures analyzed at the evaluation times (Table 4). Thus, regardless of the performance in the sessions, the ABFW phonology measures reflected improvement in the children with SSD in this study. This finding suggests that during the PROCICLOS-A sessions, the form of the dose, the planned activities, and the dose of the stimuli allowed changes in the children's performance observed in the comparison of the ABFW phonology measures between the evaluation times (Table 1).

The data obtained through the PROCICLOS-A score proved useful for understanding the difficulties and potential of each child, since different skills are worked on in the session, and each activity has a specific objective. In general, the children performed worse in the sessions in which they were presented with a new target sound and performed better in the following sessions. This shows the children's learning during the three sessions in which a target sound is worked on. In the study by Rudolph and Wendt^(24)^, one of the subjects experienced something similar when submitted to the Hodson and Paden Cycles approach^(23)^. The child in question performed better in the production of target sounds during the treatment. However, when new sounds were introduced, they clearly declined in performance. The authors suggest that, although the introduction of a new sound worsened the performance, the child improved during the treatment, indicating learning and subsequent stabilization of a new target sound in the child's phonological inventory.

The limitations of this study include the few children per group, which is an important factor in confirming the efficacy of the PROCICLOS-A approach as a function of the dose and frequency of sessions. Furthermore, it is important to conduct further research that correlates the dose of stimuli and its relationship with the dose and frequency of sessions to better understand the impact of each of these variables on SSD intervention.

## CONCLUSION

The study showed evidence of the efficacy of PROCICLOS-A, as all children improved, regardless of the initial values ​​of the measures analyzed in ABFW phonology, and the ES was medium to large between the evaluation moments. Regarding the frequency dose of the sessions, the study indicates that the total number of hours of intervention seems to interfere more than the number of sessions per week when the cumulative intensity of the intervention is maintained.

The study also presented results that show improvements in each child's performance throughout the therapeutic process, regardless of the target sound. This indicates that the PROCICLOS-A activities contribute to the learning of each sound gradually in each session, regardless of the severity at the beginning of the intervention.

These first results show that the PROCICLOS-A intervention approach is promising for the treatment of children with idiopathic phonological SSD.
