# Supplementary material for: Efficacy of the Adapted Cycles Intervention Program (PROCICLOS-A) and the influence of severity on the performance of children with Speech Sound Disorders
Source: Codas. 2025 Jul 28;37(4):e20240215. doi: 10.1590/2317-1782/e20240215en (PMC12419458; doi:10.1590/2317-1782/e20240215en)
Supplement: Translation

# Translation: Eficácia do Programa de Intervenção do Ciclos Adaptado (PROCICLOS-A) e a influência da gravidade no desempenho de crianças com Transtornos dos sons da fala

**DOI:** 10.1590/2317-1782/e20240215pt

**Authors:** Mayra Alexandra Misugi, Daniel Gomes dos Santos, Carolina Kuntz Ayub, Haydée Fiszbein Wertzner

**Affiliations:** 1 Programa de Pós-graduação em Ciências da Reabilitação, Departamento de Fisioterapia, Fonoaudiologia e Terapia Ocupacional, Faculdade de Medicina, Universidade de São Paulo – USP - São Paulo (SP), Brasil.; 2 Curso de Fonoaudiologia, Departamento de Fisioterapia, Fonoaudiologia e Terapia Ocupacional, Faculdade de Medicina da Universidade de São Paulo – USP - São Paulo (SP), Brasil.; 3 Departamento de Fisioterapia, Fonoaudiologia e Terapia Ocupacional, Faculdade de Medicina, Universidade de São Paulo – USP - São Paulo (SP), Brasil.

**Keywords:** Fonoaudiologia, Fala, Criança, Transtornos dos Sons da Fala, Intervenção

## Abstract

**Objetivo:**

Verificar a eficácia do PROCICLOS-A em crianças com TSF, em função da frequência semanal das sessões, e a influência da gravidade no desempenho das sessões.

**Método:**

Participaram oito crianças com TSF, ambos os sexos, idades entre 5:03 e 7:07 anos, alocadas em dois grupos: C1, uma vez por semana; C2, duas vezes por semana. A eficácia do PROCICLOS-A foi verificada em C1 e C2, com análise de variáveis nas provas da fonologia do teste ABFW, em três momentos de avaliação. O desempenho nas sessões foi obtido através de pontuação das atividades.

**Resultados:**

Houve diferença significante para todas as medidas, indicando melhora do desempenho nas avaliações após intervenção. Sobre a influência da dose de frequência das sessões, não houve diferença significante entre C1 e C2, nos três momentos de avaliação. O desempenho nas sessões foi analisado por meio da média dos blocos de sessões: A (sessões 1, 4, 7 e 10), B (sessões 2, 5, 8 e 11), C (sessões 3, 6, 9 e 12). Houve um menor desempenho nas sessões do bloco A e, melhor desempenho nos blocos B e C.

**Conclusão:**

O estudo mostrou evidências da eficácia do PROCICLOS-A, independentemente da dose da frequência das sessões, sugerindo que, com a manutenção da intensidade cumulativa da intervenção, o número total de horas de intervenção parece interferir mais do que o número de sessões por semana. Houve melhora no desempenho de cada criança no decorrer do programa, independentemente do som alvo e da gravidade no início da intervenção.

## INTRODUÇÃO

O Transtorno dos Sons da Fala (TSF) refere-se às dificuldades de fala que podem se manifestar em diferentes combinações entre si, quanto aos aspectos fonológicos, de percepção e de produção motora. O TSF abordado neste estudo é de causa desconhecida, tendo como principal característica o comprometimento fonológico, caracterizado por uma dificuldade cognitivo-linguística envolvendo as regras fonológicas de uma determinada língua^(1-3)^.

As crianças que apresentam TSF com comprometimento fonológico manifestam dificuldades nas representações fonológicas dos segmentos da fala, na estrutura fonotática, e/ou nos padrões de tonicidade, que são observadas na forma de utilizar, representar e organizar mentalmente o sistema fonológico da língua na qual está inserida ^(3)^. Essas crianças também podem apresentar dificuldades nas habilidades de percepção da fala e/ou processamento fonológico, que inclui as habilidades de consciência fonológica^(4,5).^

As combinações de manifestações na fala das crianças com TSF são muito variáveis, o que caracteriza a heterogeneidade entre elas. Por isso, tanto a gravidade como a inteligibilidade de fala são variáveis, o que torna fundamental a realização de avaliação fonoaudiológica com aplicação de testes que possibilitem a descrição detalhada da fala da criança e forneça indicação dos seus pontos fracos e fortes.

Para descrever a gravidade do TSF, e ainda possibilitar comparações entre a fala de diferentes crianças, algumas medidas foram propostas na literatura, entre elas a Porcentagem de Consoante Corretas (PCC)^(6)^, a Porcentagem de Consoantes Corretas Revisada (PCC-R)^(7)^ e a Densidade de Processos Fonológicos (PDI)^(8)^. Essas três medidas de gravidade têm sido usadas para indicar a eficácia de abordagens de intervenção no TSF^(9-11)^.

### Intervenção no TSF

Recomenda-se que a intervenção no TSF ocorra o mais precocemente possível, em função do risco das crianças com TSF apresentarem dificuldades na alfabetização, literacia, fracasso acadêmico, social e vocacional. Além disso, as dificuldades apresentadas podem ter consequências a longo prazo, fazendo com que as crianças não alcancem todo o seu potencial^(3,12-15)^.

Algumas abordagens no TSF, em geral, são mais voltadas à produção dos sons da fala e/ou mais ao aspecto cognitivo-linguístico (fonológico) e/ou à percepção, sendo cada uma delas aplicáveis a um tipo de TSF. Considerando essa grande divisão entre as abordagens, há várias propostas para cada uma delas, além de uma busca por classificar e entender melhor os elementos que compõem tais intervenções^(13,16,17)^. Selecionar a abordagem de intervenção no TSF de causa desconhecida depende do que foi observado na avaliação fonoaudiológica.

Outro fator essencial a ser considerado na intervenção no TSF é a intensidade e frequência necessárias para obter resultados eficazes na aplicação de uma abordagem terapêutica. Estudos têm abordado essa questão, explorando a relação entre a produção intensiva de sons e a regularidade da intervenção. Assim, a frequência semanal das sessões e a dose de estímulos oferecidos nas sessões representam a intensidade da intervenção^(9,18-20)^.

A literatura destaca que, em geral, prognósticos mais favoráveis para as crianças estão associados a uma abordagem terapêutica caracterizada por maior frequência e intensidade^(9,18,19,21,22)^. Assim, a compreensão da importância, não apenas de abordagens terapêuticas eficazes, mas também da necessidade de uma abordagem intensiva e consistente para otimizar os resultados no desenvolvimento da fala em crianças, são fundamentais.

Em estudo de revisão sistemática, em que foi verificada a eficácia e eficiência das abordagens disponíveis, sugere-se que algumas delas carecem de detalhes e esclarecimentos, pois são ambíguas em relação à aplicação e à forma como são entregues às crianças pelos fonoaudiólogos^(17)^.

Além disso, também destacam a necessidade de estudos randomizados que investiguem a eficácia, a frequência das sessões e a dose de estímulos por sessão.

### Abordagem dos ciclos

Dentre as abordagens que buscam intervir no TSF, destaca-se a abordagem dos ciclos, proposta por Hodson e Paden^(23)^, a qual fundamenta-se na interação dos processos cognitivo-linguísticos, perceptivos e de produção da fala. Nesta abordagem, é essencial que as prioridades para a intervenção sejam determinadas considerando os processos fonológicos que a criança apresentou na avaliação inicial e a estimulabilidade dos sons ausentes do inventário fonético.

A intervenção é desenvolvida em ciclos, sendo que cada ciclo corresponde ao período no qual são tratados os processos fonológicos que mais afetam a inteligibilidade da fala da criança. Cada som-alvo do processo fonológico selecionado é abordado por 60 minutos, quando ocorre a troca para outro som-alvo. Assim, em um ciclo, vários sons são abordados. Além disso, são propostas atividades específicas que envolvem o uso de pistas semânticas, tátil-cinestésicas, auditivas e visuais, conduzindo a criança por meio de estratégias que favorecem o desenvolvimento das habilidades da fala e da linguagem.

Um estudo de Rudolph e Wendt^(24)^, nos mostra que a abordagem de ciclos proposta por Hodson e Paden^(23)^ é eficaz, na medida em que combina uma seleção adequada dos processos fonológicos de cada criança, tratados em formato cíclico, por meio de atividades que trabalham o *input* auditivo e a prática da produção do som alvo. Dessa forma, a abordagem resulta em uma melhora no que se refere às trocas na fala da criança, proporcionando inclusive, generalização de sons não trabalhados. Ainda, o estudo sugere que é possível aplicar variações à abordagem, tais como: variação da intensidade, estratégias para trabalhar o som alvo, além da ordem em que cada som alvo será trabalhado, como formas de se alcançar melhores desempenhos das crianças.

Outro estudo, de Cabbage et al.^(25)^, que investiga a respeito da escolha dos terapeutas, diante das diferentes abordagens terapêuticas existentes, mostra que, dentre as mais diversas intervenções, as que envolvem uma abordagem articulatória tradicional, como a abordagem de ciclos de Hodson e Paden^(23)^, são as mais escolhidas por fonoaudiólogos. Os resultados mostraram que não somente os entrevistados utilizaram a abordagem dos ciclos para atendimento, como também costumavam combiná-la com outras abordagens, de acordo com cada caso e necessidade.

A partir da proposta original dos Ciclos por Hodson e Paden^(23)^, foi elaborada uma adaptação para os falantes do Português Brasileiro por Wertzner e Pagan-Neves^(26)^. Na abordagem dos Ciclos Adaptada as autoras propõem ciclos de sete sessões, nas quais o processo fonológico e o som alvo estimulável são selecionados a partir da avaliação inicial. Nas sessões de 1 a 3, são realizadas atividades de bombardeamento auditivo do som alvo, colocação fonêmica, reforço articulatório do som alvo, atividade de discriminação auditiva e atividades com pares mínimos envolvendo o som alvo escolhido. Nas sessões 4 a 7, acrescentam-se atividades de metafonologia e, ao final da 7ª sessão, é aplicada a prova de verificação para observar a generalização ocorrida^(26)^.

Neste estudo, é apresentado o Programa do Ciclos Adaptado (PROCICLOS-A), no qual cada ciclo é composto por 12 sessões. Ao longo das sessões são abordados dois processos fonológicos e um total de quatro sons alvos. O desempenho da criança em cada atividade das sessões é pontuado, permitindo observar além do som alvo mais fácil e mais difícil, as habilidades que representam maior desafio para a criança. Pretende-se responder às seguintes questões: as crianças que obtiveram menores valores das medidas na Fonologia ABFW na avaliação inicial, têm melhor desempenho nas sessões do PROCICLOS-A? O número de sessões por semana interfere na melhora das crianças?

O objetivo do estudo foi verificar a eficácia do PROCICLOS-A em crianças com TSF, em função da frequência semanal das sessões, e a influência da gravidade no desempenho das sessões.

## MÉTODO

### Sujeitos

O estudo foi aprovado pelo Comitê de Ética em Pesquisa (CAAE 87068318.2.0000.0065). Trata-se de um estudo prospectivo, experimental, randomizado. Os responsáveis assinaram o Termo de Consentimento Livre e Esclarecido e as crianças assinaram o Termo de Assentimento. O presente estudo é parte de uma pesquisa de intervenção, em que as crianças são distribuídas de forma randomizada em três diferentes programas de intervenção e com diferentes frequências semanais.

A randomização foi feita eletronicamente, usando a função “aleatória” do programa Excel, pela última autora do estudo, e foi elaborada para que houvesse uma distribuição equilibrada entre as três intervenções. No presente estudo, participaram oito crianças, que foram alocadas no PROCICLOS-A, em dois grupos distintos, de acordo com a frequência das sessões, de forma randomizada: o primeiro grupo, com quatro crianças, foi submetido à intervenção com frequência de uma vez por semana (C1); o segundo grupo, também com quatro crianças, à intervenção com frequência duas vezes por semana (C2).

A avaliação fonoaudiológica para o diagnóstico foi realizada em um laboratório de pesquisa ligado a uma universidade. Os protocolos utilizados foram: ABFW Teste de linguagem infantil nas áreas de fonologia, vocabulário, fluência e pragmática^(27)^, Teste de Sensibilidade Fonológica – Auditivo (TSF-A)^(28,29)^, *Comprehensive Test of Phonological Processing* (CTOPP – Nomeação Rápida)^(30)^, Discriminação Auditiva, protocolo não publicado elaborado no laboratório de pesquisa, que inclui os contrastes de ponto, modo e vozeamento, tanto em sílabas isoladas como em dissílabas, Prova de Inconsistência de Fala^(31)^, Avaliação Miofuncional Orofacial com Escores (AMIOFE)^(32)^, e o Teste de Estimulabilidade dos Sons da Fala (TESF)^(33)^.

As oito crianças com TSF, de ambos os sexos, tinham idades entre 5:03 e 7:07 anos. Foram critérios de inclusão: obter na avaliação inicial a Porcentagem de Consoantes Corretas revisado (PCC-R)^(7)^ abaixo de 93,4%^(34)^ na prova de nomeação da Fonologia do ABFW Teste de linguagem infantil nas áreas de fonologia, vocabulário, fluência e pragmática (Fonologia ABFW)^(35)^; manifestar ao menos um processo fonológico com ocorrência mínima de 25% em uma das duas provas da Fonologia ABFW; ter avaliação audiológica dentro dos limites de normalidade (abaixo de 20 dB nas frequências de 500, 1000, 2000, e 4000 Hz); QI não-verbal dentro dos limites de normalidade^(36)^, realizado por uma profissional psicóloga; ter como língua materna o português brasileiro.

### Procedimento

#### Avaliação para a linha de base e reavaliações

As crianças foram avaliadas em três momentos diferentes. O primeiro momento, correspondente à avaliação que antecedeu o início da intervenção terapêutica, chamada de avaliação de linha de base (A1); a segunda avaliação (A2), que ocorreu após o término da aplicação do PROCICLOS-A; e a terceira avaliação (A3), realizada 6 semanas após a segunda avaliação, em que as crianças ficaram sem intervenção. Para a linha de base (A1) e reavaliações, foram consideradas as provas de nomeação e imitação da fonologia ABFW^(35)^, para as quais foram calculadas as medidas Porcentagem de Consoantes Corretas (PCC)^(6)^, Porcentagem de Consoantes Corretas-Revisada (PCC-R)^(7)^, *Phonological Density Index* (PDI)^(8)^ e o número de processos fonológicos (NPF) com ocorrência > 25%. As avaliações foram gravadas em vídeo, e suas respectivas análises foram feitas por dois fonoaudiólogos, com concordância de 85%. Os resultados das avaliações foram analisados para verificar a eficácia do PROCICLOS-A.

A avaliação para diagnóstico de cada criança, bem como as reavaliações ao longo, e ao término dos programas de intervenção, foram realizadas por uma fonoaudióloga, pós-graduanda, cega ao procedimento de intervenção do estudo. Da mesma forma, o fonoaudiólogo que aplicou o PROCICLOS-A era cego à avaliação, sendo que o pesquisador sênior (quarto autor) selecionou os sons alvos a serem abordados nas sessões.

Os critérios para a seleção dos processos fonológicos, assim como a seleção dos sons alvos trabalhados, foram: preferencialmente processos fonológicos ocorrência com ocorrência >25%, que não eram mais esperados para a idade da criança, e que comprometessem sua inteligibilidade de fala. Ainda, foi levada em consideração a estimulabilidade dos sons, tendo sido escolhidos aqueles que foram estimuláveis.

### PROCICLOS-A

O PROCICLOS-A^(37)^ é uma adequação da Abordagem de Ciclos Adaptado^(26)^ que, por sua vez, foi elaborado como uma modificação da abordagem de Ciclos de Hodson e Paden^(23)^. O PROCICLOS-A consta de 12 sessões de intervenção, com duração média de 50 minutos, que têm como objetivo desenvolver a percepção auditiva, a propriocepção dos movimentos articulatórios, as regras fonológicas e a consciência fonológica.

Da mesma forma que na Abordagem do Ciclos^(23)^ o PROCICLOS-A considera que o domínio dos sons e das regras fonológicas é gradual durante a aquisição, a intervenção no TSF deve expor a criança de forma gradativa aos sons da fala. Assim, novos sons alvos são introduzidos no tratamento antes que os anteriores sejam dominados. São selecionados dois processos fonológicos e dois sons alvos em cada um deles, para serem trabalhados durante as 12 sessões. O critério para seleção dos processos fonológicos inclui aqueles que mais comprometem a inteligibilidade de fala e que estão de acordo com o esperado para a idade; em relação aos sons alvos de cada processo fonológico são escolhidos aqueles que são estimuláveis. As sessões de 1 a 6 abordam os dois sons-alvos do primeiro processo fonológico selecionado, sendo que as sessões de 1 a 3 referem-se ao primeiro e as de 4 a 6 ao segundo. As sessões de 7 a 12 tratam do segundo processo fonológico, sendo o primeiro som-alvo abordado nas sessões de 7 a 9, e o segundo som alvo nas sessões de 10 a 12, como mostra a Figura 1.

**Figura 1:** Esquema da abordagem PROCICLOS-A

**Legenda:** Bloco: A (sessões 1, 4, 7 e 10), B (sessão 2, 5, 8 e 11) e C (sessões 3, 6, 9 e 12)

Seis tipos de atividades são desenvolvidas em cada uma das 12 sessões da intervenção fonoaudiológica, abrangendo os sons-alvo selecionados para cada uma das sessões. As atividades selecionadas foram: Bombardeamento Auditivo (no início e final da sessão); Colocação do Som alvo; Reconhecimento do som alvo e Discriminação Auditiva com Pares Mínimos; Atividades com Pares Mínimos para compreensão da Regra; Treino em Palavras com o som alvo em posição inicial, medial e final; e Atividades de Consciência Fonológica (Quadro 1). As atividades foram planejadas de forma que as sessões tenham uma duração média de 50 minutos, e que a criança tenha a possibilidade de produzir o som alvo pelo menos 100 vezes durante cada sessão.

**Quadro 1:** Atividades das sessões do PROCICLOS-A

1. Bombardeamento Auditivo	A fonoaudióloga lê pausadamente as palavras dissílabas que iniciam com os sons-alvo do processo que está sendo trabalhado. A criança deve ouvir atentamente estas palavras. Duração média da atividade: 1 minuto.
2. Apresentação e Produção Articulatória do Som Alvo	Essa atividade tem como objetivo auxiliar a criança a produzir os sons, por meio de pistas facilitadoras multimodais, ou seja, pistas auditivas, visuais e táteis. Duração média da atividade: 10 minutos.
3. A) Reconhecimento Auditivo; B) Discriminação Auditiva	No Reconhecimento Auditivo, a criança deve manifestar o reconhecimento do som alvo trabalhado sempre que o ouvir. Na Discriminação Auditiva, a criança deve identificar qual o par igual e qual o par diferente. Duração média das atividades: 5 minutos.
4. Compreensão da Regra com Pares Mínimos	A atividade tem como objetivo auxiliar a criança na compreensão e utilização da regra fonológica. Para cada som alvo de um processo fonológico foram selecionados 3 a 5 pares mínimos por oposição mínima ou por oposição máxima. Tempo médio da atividade: de 15 a 20 minutos.
5. Treino em palavras com o som alvo em posição inicial, medial e final	São utilizadas estratégias para trabalhar a correta produção do som alvo quando em posição de sílaba inicial, medial e final. Tempo médio da estratégia: de 15 a 20 minutos.
6. Consciência Fonológica	O objetivo é estimular a consciência fonológica, por meio das habilidades de rima, aliteração e segmentação silábica. Duração média da estratégia: 10 minutos.
7. Bombardeamento Auditivo	A fonoaudióloga lê pausadamente as palavras dissílabas que iniciam com os sons-alvo do processo que está sendo trabalhado. A criança deve ouvir atentamente estas palavras. Duração média da atividade: 1 minuto.

Para cada atividade, foram elaboradas estratégias diferentes, sendo que, para sua análise nas 12 sessões, considerou-se o seu desempenho por bloco de sessões: A, B e C. O agrupamento A corresponde às sessões de números 1, 4, 7 e 10, referentes à introdução de um novo som alvo. O agrupamento B corresponde às sessões de números 2, 5, 8 e 11, e se referem às sessões mediais de cada som alvo. O agrupamento C corresponde às sessões de números 3, 6, 9 e 12, e referem-se às últimas sessões trabalhadas com cada som alvo escolhido.

### Pontuação do desempenho nas sessões

Para analisar o desempenho em cada uma das 12 sessões, bem como nas atividades do PROCICLOS-A, foi elaborado um sistema de pontuação, no qual é atribuído 2 pontos para o acerto, 1 ponto para acerto parcial e 0 para erro. Destaca-se que foi considerado como acerto parcial a ocorrência de produção do som alvo com distorção, ou um acerto na segunda tentativa de produção. São pontuadas as atividades 2, 3, 4, 5 e 6 (Quadro 1). Como os estímulos para cada processo fonológico e som-alvo variam, o valor total esperado em cada sessão também varia.

Para registrar o desempenho de cada criança, foram criadas planilhas no Excel^®^ para cada som-alvo nas quais estão indicados os valores esperados para cada atividade e são anotados os valores obtidos. No presente estudo, a pontuação das sessões de cada criança foi realizada pelos dois primeiros autores e calculado a concordância que se manteve acima de 80% para todas as sessões das oito crianças do estudo.

### Análise estatística

A análise descritiva dos dados incluiu o cálculo de medidas de tendência central e dispersão. Para as comparações intergrupos foi utilizado o teste U de Mann-Whitney e para comparações intragrupos foi realizada a análise de variância univariada (ANOVA) de Friedman com análise post hoc utilizando o teste de postos sinalizados de Wilcoxon com correção de Bonferroni para múltiplas comparações. As análises de correlação foram feitas por meio do teste de correlação de Spearman. Devido ao baixo número amostral, todas as hipóteses foram testadas por meio de procedimentos não-paramétricos e, para além do nível de significância fixado em 5% (p ≤ 0,05), utilizou-se também a mensuração do tamanho do efeito por meio do cálculo do coeficiente r^(38)^ para complementar a interpretação dos resultados. Para interpretar o tamanho do efeito (TE), sugere-se utilizar a classificação proposta por Cohen^(39)^: pequeno (entre |0,200| e |0,499|); médio (entre |0,500| e |0,799|); e grande (acima de |0,800|).

## RESULTADOS

Os resultados do estudo incluem a verificação da eficácia do PROCICLOS-A, comparando os três momentos de avaliação para cada um dos grupos C1 e C2; a análise da Influência da dose de frequência das sessões e o desempenho nas sessões do PROCICLOS-A.

No Quadro 2, encontra-se a descrição das crianças participantes do estudo, de acordo com os grupos, incluindo os processos fonológicos e sons alvos abordados no PROCICLOS-A.

**Quadro 2:** Dados Descritivos das crianças do estudo na avaliação inicial

Grupos	Sujeitos	Idade	Sexo	Processos Fonológicos Alvos	Sons Alvo
C1	Criança 1	5:11	Masculino	SL, FV	/ɾ/, /ʎ/, /k/, /g/
	Criança 2	6:08	Feminino	EF, EP	/v/, /z/, /b/, /d/
	Criança 3	7:07	Masculino	FV, FP	/k/, /g/, /ʃ/, /ʒ/
	Criança 4	5:06	Masculino	FV, EF	/k/, /g/, /v/, /z/
C2	Criança 5	6:00	Feminino	FP, SEC	/ʃ/, /ʒ/, Enc _R
	Criança 6	5:03	Masculino	FP, EF	/ʃ/, /ʒ/, /v/, /z/
	Criança 7	5:03	Masculino	FP, EF	/ʃ/, /ʒ/, /v/, /z/
	Criança 8	6:05	Masculino	SEC	Enc /l/ Enc/ɾ/

**Legenda:** C1: grupo uma vez na semana; C2: grupo duas vezes na semana; FV: Frontalização de Velares; FP: Frontalização de Palatal; SL: Simplificação de Líquidas; SEC: Simplificação de Encontro Consonantal; EP: Ensurdecimento de Plosiva; EF: Ensurdecimento de Fricativa

### Eficácia do PROCICLOS-A

Para comprovar a eficácia no PROCICLOS-A em cada um dos grupos, foi realizada a comparação das variáveis PCC, PCC-R, PDI e NPF nos três momentos de avaliação. Os resultados apresentados nas Tabelas 1 e 2, mostram que houve diferença significante para todas as medidas, com exceção de NPF-Nomeação, para C1 e C2, e PCC-Imitação para C2. Destaca-se que o desempenho melhorou nas avaliações após a intervenção.

**Tabela 1:** Valores descritivos e análise comparativa dos momentos de acordo com o grupo e a tarefa

Grupo	Medida	Momento	n	Nomeação						
				Média	DP	Mediana	Mín.	Máx.	P	T.E.
C1	NPF	A1	4	4,25	0,96	4,50	3,00	5,00	0,093	0,544^†^
		A2	4	2,75	2,22	3,00	0,00	5,00		
		A3	4	2,50	1,73	2,50	1,00	4,00		
	PCC	A1	4	67,75	13,72	66,10	54,40	84,40	0,009*	0,925^†^
		A2	4	83,30	12,16	83,30	70,00	96,60		
		A3	4	84,43	12,76	85,00	70,00	97,70		
	PCC-R	A1	4	68,88	12,83	66,65	57,80	84,40	0,009*	0,925^†^
		A2	4	83,78	11,84	84,25	70,00	96,60		
		A3	4	84,98	12,42	86,10	70,00	97,70		
	PDI	A1	4	0,82	0,34	0,89	0,41	1,10	0,005*	0,925^†^
		A2	4	0,50	0,39	0,51	0,08	0,88		
		A3	4	0,42	0,37	0,37	0,06	0,88		
C2	NPF	A1	4	4,00	2,31	4,00	2,00	6,00	0,074	0,465^†^
		A2	4	3,25	3,20	3,50	0,00	6,00		
		A3	4	2,00	1,83	2,00	0,00	4,00		
	PCC	A1	4	66,68	12,31	62,25	57,80	84,40	0,042*	0,720^†^
		A2	4	75,80	15,30	73,30	62,20	94,40		
		A3	4	79,95	12,31	75,50	71,10	97,70		
	PCC-R	A1	4	71,68	15,40	71,65	57,80	85,60	0,005*	1,000^†^
		A2	4	79,73	18,35	80,00	62,20	96,70		
		A3	4	86,88	13,42	88,25	72,20	98,80		
	PDI	A1	4	0,76	0,40	0,77	0,38	1,12	0,005*	1,000^†^
		A2	4	0,53	0,49	0,53	0,09	1,00		
		A3	4	0,36	0,36	0,32	0,05	0,74		
Total	NPF	A1	8	4,12	1,64	4,50	2,00	6,00	0,002*	0,484^†^
		A2	8	3,00	2,56	3,00	0,00	6,00		
		A3	8	2,25	1,67	2,00	0,00	4,00		
	PCC	A1	8	67,21	12,08	62,25	54,40	84,40	< 0,001*	0,813^†^
		A2	8	79,55	13,41	79,40	62,20	96,60		
		A3	8	82,19	11,85	78,30	70,00	97,70		
	PCC-R	A1	8	70,27	13,21	66,65	57,80	85,60	< 0,001*	0,958^†^
		A2	8	81,75	14,46	84,25	62,20	96,70		
		A3	8	85,93	12,01	86,10	70,00	98,80		
	PDI	A1	8	0,79	0,34	0,89	0,38	1,12	< 0,001*	0,958^†^
		A2	8	0,51	0,41	0,51	0,08	1,00		
		A3	8	0,39	0,34	0,37	0,05	0,88		
Grupo	Medida	Momento	n	Imitação						
				Média	DP	Mediana	Mín.	Máx.	p	T.E.
C1	NPF	A1	4	4,00	1,41	4,50	2,00	5,00	0,037*
		A2	4	2,25	2,06	2,50	0,00	4,00	
		A3	4	2,50	1,73	2,50	1,00	4,00	
	PCC	A1	4	67,50	5,98	67,75	60,70	73,80	0,009*	0,925^†^
		A2	4	82,63	12,18	82,20	68,90	97,20		
		A3	4	86,90	10,62	87,80	74,80	97,20		
	PCC-R	A1	4	68,68	5,81	70,10	60,70	73,80	0,009*	0,925^†^
		A2	4	82,63	12,18	82,20	68,90	97,20		
		A3	4	87,35	9,96	87,80	76,60	97,20		
	PDI	A1	4	0,87	0,15	0,85	0,72	1,07	0,009*	0,925^†^
		A2	4	0,49	0,35	0,52	0,07	0,85		
		A3	4	0,36	0,28	0,37	0,07	0,61		
C2	NPF	A1	4	4,00	2,45	3,50	2,00	7,00	0,037*	0,786^†^
		A2	4	1,75	2,06	1,50	0,00	4,00		
		A3	4	1,75	2,06	1,50	0,00	4,00		
	PCC	A1	4	66,88	10,02	63,55	58,90	81,50	0,069	0,625^†^
		A2	4	83,70	11,46	83,20	71,00	97,40		
		A3	4	82,93	9,08	79,45	76,60	96,20		
	PCC-R	A1	4	71,78	12,86	72,05	58,90	84,10	0,042*	0,720^†^
		A2	4	86,43	13,41	88,30	71,00	98,10		
		A3	4	88,30	10,36	88,75	77,60	98,10		
	PDI	A1	4	0,78	0,35	0,78	0,44	1,13	0,042*	0,720^†^
		A2	4	0,37	0,36	0,32	0,05	0,79		
		A3	4	0,31	0,30	0,29	0,05	0,62		
Total	NPF	A1	8	4,00	1,85	4,50	2,00	7,00	< 0,001*	0,841^†^
		A2	8	2,00	1,93	2,00	0,00	4,00		
		A3	8	2,13	1,81	2,00	0,00	4,00		
	PCC	A1	8	67,19	7,65	64,50	58,90	81,50	< 0,001*	0,715^†^
		A2	8	83,16	10,96	82,70	68,90	97,40		
		A3	8	84,91	9,39	81,30	74,80	97,20		
	PCC-R	A1	8	70,22	9,39	70,10	58,90	84,10	< 0,001*	0,813†
		A2	8	84,53	12,04	83,15	68,90	98,10		
		A3	8	87,83	9,42	87,80	76,60	98,10		
	PDI	A1	8	0,83	0,25	0,85	0,44	1,13	< 0,001*	0,813^†^
		A2	8	0,43	0,34	0,46	0,05	0,85		
		A3	8	0,33	0,27	0,33	0,05	0,62		

* valor estatisticamente significante no nível de 5% (p ≤ 0,05);

† Tamanho do efeito maior ou igual a moderado/médio

**Legenda:** DP: Desvio padrão; Mín.: Mínimo; Máx.: Máximo; T.E.: Tamanho do efeito

Analisou-se também o TE, no qual foram observados resultados que variaram entre moderado e grande, para todas as medidas, nos diferentes momentos de avaliação, tanto para C1 quanto para C2, indicando um melhor desempenho das crianças após aplicação do PROCICLOS-A.

### Análise da Influência da Dose de Frequência das Sessões

Para análise da influência da dose de frequência das sessões, foram analisados o desempenho obtido por C1 e C2, nos três momentos de avaliação. Na Tabela 2, observa-se que não houve diferença significante entre C1 e C2 em relação às medidas estudadas NPF, PCC, PCC-R, PDI nas provas da Fonologia ABFW (nomeação e imitação) nos três momentos de avaliação. No entanto, analisando o TE, verificou-se valores de moderado a grande para as medidas de PCC-Nomeação para C1 em A2, e de NPF-Imitação para C2 em A3. As análises realizadas indicam que C1 e C2 tiveram desempenhos semelhantes em A1 e, após o PROCICLOS-A, observou-se TE grande para C1, e TE moderado para C2.

**Tabela 2:** Valores descritivos e análise comparativa das medidas da Fonologia ABFW entre C1 e C2, de acordo com o momento de avaliação

Momento	Medida	Grupo	n	Nomeação						
				Média	DP	Mediana	Mín.	Máx.	P	T.E.
A1	NPF	C1	4	4,25	0,96	4,50	3,00	5,00	> 0,999	0,000
		C2	4	4,00	2,31	4,00	2,00	6,00		
	PCC	C1	4	67,75	13,72	66,10	54,40	84,40	> 0,999	0,000
		C2	4	66,68	12,31	62,25	57,80	84,40		
	PCC-R	C1	4	68,88	12,83	66,65	57,80	84,40	0,829	0,156
		C2	4	71,68	15,40	71,65	57,80	85,60		
	PDI	C1	4	0,82	0,34	0,89	0,41	10,10	0,971	0,051
		C2	4	0,76	0,40	0,77	0,38	10,12		
A2	NPF	C1	4	2,75	2,22	3,00	0,00	5,00	0,743	0,155
		C2	4	3,25	3,20	3,50	0,00	6,00		
	PCC	C1	4	83,30	12,16	83,30	70,00	96,60	0,486	0,306^†^
		C2	4	75,80	15,30	73,30	62,20	94,40		
	PCC-R	C1	4	83,78	11,84	84,25	70,00	96,60	0,886	0,102
		C2	4	79,73	18,35	80,00	62,20	96,70		
	PDI	C1	4	0,50	0,39	0,51	0,08	0,88	0,686	0,204
		C2	4	0,53	0,49	0,53	0,09	1,00		
A3	NPF	C1	4	2,50	1,73	2,50	1,00	4,00	0,743	0,215
		C2	4	2,00	1,83	2,00	0,00	4,00		
	PCC	C1	4	84,43	12,76	85,00	70,00	97,70	0,971	0,051
		C2	4	79,95	12,31	75,50	71,10	97,70		
	PCC-R	C1	4	84,98	12,42	86,10	70,00	97,70	0,743	0,154
		C2	4	86,88	13,42	88,25	72,20	98,80		
	PDI	C1	4	0,42	0,37	0,37	0,06	0,88	0,600	0,205
		C2	4	0,36	0,36	0,32	0,05	0,74		
Momento	Medida	Grupo	n	Imitação						
				Média	DP	Mediana	Mín.	Máx.	p	T.E.
A1	NPF	C1	4	4,00	1,41	4,50	2,00	5,00	> 0,999	0,000
		C2	4	4,00	2,45	3,50	2,00	7,00		
	PCC	C1	4	67,50	5,98	67,75	60,70	73,80	0,743	0,154
		C2	4	66,88	10,02	63,55	58,90	81,50		
	PCC-R	C1	4	68,68	5,81	70,10	60,70	73,80	0,886	0,102
		C2	4	71,78	12,86	72,05	58,90	84,10		
	PDI	C1	4	0,87	0,15	0,85	0,72	1,07	0,886	0,102
		C2	4	0,78	0,35	0,78	0,44	1,13		
A2	NPF	C1	4	2,25	2,06	2,50	0,00	4,00	0,743	0,215
		C2	4	1,75	2,06	1,50	0,00	4,00		
	PCC	C1	4	82,63	12,18	82,20	68,90	97,20	0,686	0,204
		C2	4	83,70	11,46	83,20	71,00	97,40		
	PCC-R	C1	4	82,63	12,18	82,20	68,90	97,20	0,543	0,257
		C2	4	86,43	13,41	88,30	71,00	98,10		
	PDI	C1	4	0,49	0,35	0,52	0,07	0,85	0,686	0,204
		C2	4	0,37	0,36	0,32	0,05	0,79		
A3	NPF	C1	4	2,50	1,73	2,50	1,00	4,00	0,400	0,318†
		C2	4	1,75	2,06	1,50	0,00	4,00		
	PCC	C1	4	86,90	10,62	87,80	74,80	97,20	0,743	0,154
		C2	4	82,93	9,08	79,45	76,60	96,20		
	PCC-R	C1	4	87,35	9,96	87,80	76,60	97,20	0,743	0,154
		C2	4	88,30	10,36	88,75	77,60	98,10		
	PDI	C1	4	0,36	0,28	0,37	0,07	0,61	0,743	0,154
		C2	4	0,31	0,30	0,29	0,05	0,62		

Teste de postos sinalizados de Wilcoxon

† Tamanho do efeito maior ou igual a moderado/médio

**Legenda**: DP: Desvio padrão; Mín.: Mínimo; Máx.: Máximo; T.E.: Tamanho do efeito

### Desempenho nas sessões do PROCICLOS-A

Para análise do desempenho médio das crianças nas sessões, foi empregado o sistema de pontuação para cada uma das 12 sessões, bem como nas atividades realizadas. Na Tabela 3 são apresentadas as medidas PCC, PCC-R e PDI na avaliação inicial para cada criança, e o desempenho médio nas 12 sessões. O desempenho médio foi obtido por meio das pontuações de cada sessão, e a partir das pontuações foi calculado o desempenho em porcentagem e realizada a média do desempenho nas 12 sessões do PROCICLOS-A para cada criança.

**Tabela 3:** Valores das medidas da fonologia ABFW na A1 e desempenho médio das crianças nas 12 sessões do PROCICLOS-A

Grupo	Sujeitos	PDI		PCC (%)		PCC-R (%)		Desempenho médio nas 12 sessões
		A1		A1		A1		
		N	I	N	I	N	I	
C1	Criança 1	0,68	0,82	73,3	71,0	74,4	72,0	73%
	Criança 2	1,09	0,87	54,4	64,5	57,8	68,2	86%
	Criança 3	0,41	0,72	84,4	73,8	84,4	73,8	85%
	Criança 4	1,1	1,07	58,9	60,7	58,9	60,7	68%
C2	Criança 5	0,38	0,44	65,6	64,5	85,6	84,1	72%
	Criança 6	1,09	1,13	58,9	58,9	58,9	58,9	83%
	Criança 7	1,12	1,03	57,8	62,6	57,8	62,6	68%
	Criança 8	0,44	0,52	84,4	81,5	84,4	81,5	64%

**Legenda:** A1: avaliação inicial. N: nomeação. I: imitação

A Figura 2 mostra as medidas de tendência central e dispersão do desempenho das sessões agrupadas em blocos de sessões A (sessões 1, 4, 7 e 10), B (sessões 2, 5, 8 e 11), C (sessões 3, 6, 9 e 12) e o total das sessões, por meio da análise descritiva dos dados. As crianças obtiveram um menor desempenho nas sessões do bloco A, que correspondem à apresentação de um novo som alvo. Observando o desempenho total, o grupo C1 demonstrou maior desempenho médio (78%) quando comparado com o grupo C2 (72%).

**Figura 2:** Medidas de tendência central e dispersão do desempenho nas sessões

O desempenho médio de cada criança, para cada uma das 12 sessões, é apresentado na Figura 3. As crianças apresentam um menor desempenho nas sessões do bloco A, e ocorre a melhora do desempenho nas sessões dos blocos B e C.

**Figura 3:** Desempenho médio de cada criança nas 12 sessões do PROCICLOS-A

Foi analisada a correlação de Spearman entre o desempenho médio em cada um dos blocos de sessões A, B e C e cada uma das medidas das provas de fonologia ABFW obtidos nas avaliações A1, A2 e A3 (Tabela 4). Não foram observadas correlações estatisticamente significantes para todas as análises.

**Tabela 4:** Correlação entre o desempenho nos blocos de sessões com as medidas da fonologia ABFW de acordo com o momento da avaliação

		Ses.	Tar.		C1 (n = 4)					
					A1	A2	A3	A2-A1	A3-A1	A3-A2
	NPF	A	N	Coef.	-0,105	0,200	0,000	0,200	0,105	-0,447^†^
				p	0,895	0,800	> 0,999	0,800	0,895	0,553
			I	Coef.	-0,105	-0,105	0,000	0,105	0,258	NC
				p	0,895	0,895	> 0,999	0,895	0,742	NC
		B	N	Coef.	-0,105	0,200	0,000	0,200	0,105	-0,447^†^
				p	0,895	0,800	> 0,999	0,800	0,895	0,553
			I	Coef.	-0,105	-0,105	0,000	0,105	0,258	NC
				p	0,895	0,895	> 0,999	0,895	0,742	NC
		C	N	Coef.	-0,105	0,200	0,000	0,200	0,105	-0,447^†^
				p	0,895	0,800	> 0,999	0,800	0,895	0,553
			I	Coef.	-0,105	-0,105	0,000	0,105	0,258	NC
				p	0,895	0,895	> 0,999	0,895	0,742	NC
		Total	N	Coef.	-0,105	0,200	0,000	0,200	0,105	-0,447^†^
				p	0,895	0,800	> 0,999	0,800	0,895	0,553
			I	Coef.	-0,105	-0,105	0,000	0,105	0,258	NC
				p	0,895	0,895	> 0,999	0,895	0,742	NC
	PCC	A	N	Coef.	-0,200	0,400^†^	0,400^†^	0,800^†^	0,800^†^	0,400^†^
				p	0,800	0,600	0,600	0,200	0,200	0,600
			I	Coef.	0,400^†^	-0,200	-0,200	-0,400^†^	-0,200	0,000
				p	0,600	0,800	0,800	0,600	0,800	> 0,999
		B	N	Coef.	-0,200	0,400^†^	0,400^†^	0,800^†^	0,800^†^	0,400^†^
				p	0,800	0,600	0,600	0,200	0,200	0,600
			I	Coef.	0,400^†^	-0,200	-0,200	-0,400^†^	-0,200	0,000
				p	0,600	0,800	0,800	0,600	0,800	> 0,999
		C	N	Coef.	-0,200	0,400^†^	0,400^†^	0,800^†^	0,800^†^	0,400^†^
				p	0,800	0,600	0,600	0,200	0,200	0,600
			I	Coef.	0,400^†^	-0,200	-0,200	-0,400^†^	-0,200	0,000
				p	0,600	0,800	0,800	0,600	0,800	> 0,999
		Total	N	Coef.	-0,200	0,400^†^	0,400^†^	0,800^†^	0,800^†^	0,400^†^
				p	0,800	0,600	0,600	0,200	0,200	0,600
			I	Coef.	0,400^†^	-0,200	-0,200	-0,400^†^	-0,200	0,000
				p	0,600	0,800	0,800	0,600	0,800	> 0,999
	PCC-R	A	N	Coef.	-0,200	0,400^†^	0,400^†^	0,800^†^	0,800^†^	0,400^†^
				p	0,800	0,600	0,600	0,200	0,200	0,600
			I	Coef.	0,400^†^	-0,200	-0,200	-0,400^†^	-0,200	0,400^†^
				p	0,600	0,800	0,800	0,600	0,800	0,600
		B	N	Coef.	-0,200	0,400^†^	0,400^†^	0,800^†^	0,800^†^	0,400^†^
				p	0,800	0,600	0,600	0,200	0,200	0,600
			I	Coef.	0,400^†^	-0,200	-0,200	-0,400^†^	-0,200	0,400^†^
				p	0,600	0,800	0,800	0,600	0,800	0,600
		C	N	Coef.	-0,200	0,400^†^	0,400^†^	0,800^†^	0,800^†^	0,400^†^
				p	0,800	0,600	0,600	0,200	0,200	0,600
			I	Coef.	0,400^†^	-0,200	-0,200	-0,400^†^	-0,200	0,400^†^
				p	0,600	0,800	0,800	0,600	0,800	0,600
		Total	N	Coef.	-0,200	0,400^†^	0,400^†^	0,800^†^	0,800^†^	0,400^†^
				p	0,800	0,600	0,600	0,200	0,200	0,600
			I	Coef.	0,400^†^	-0,200	-0,200	-0,400^†^	-0,200	0,400^†^
				p	0,600	0,800	0,800	0,600	0,800	0,600
	PDI	A	N	Coef.	-0,400^†^	-0,400^†^	-0,400^†^	-0,400^†^	-0,800^†^	-0,800^†^
				p	0,600	0,600	0,600	0,600	0,200	0,200
			I	Coef.	-0,400^†^	0,200	0,200	0,200	0,400^†^	-0,400^†^
				p	0,600	0,800	0,800	0,800	0,600	0,600
		B	N	Coef.	-0,400^†^	-0,400^†^	-0,400^†^	-0,400^†^	-0,800^†^	-0,800^†^
				p	0,600	0,600	0,600	0,600	0,200	0,200
			I	Coef.	-0,400^†^	0,200	0,200	0,200	0,400^†^	-0,400^†^
				p	0,600	0,800	0,800	0,800	0,600	0,600
		C	N	Coef.	-0,400^†^	-0,400^†^	-0,400^†^	-0,400^†^	-0,800^†^	-0,800^†^
				p	0,600	0,600	0,600	0,600	0,200	0,200
			I	Coef.	-0,400^†^	0,200	0,200	0,200	0,400^†^	-0,400^†^
				p	0,600	0,800	0,800	0,800	0,600	0,600
		Total	N	Coef.	-0,400^†^	-0,400^†^	-0,400^†^	-0,400^†^	-0,800^†^	-0,800^†^
				P	0,600	0,600	0,600	0,600	0,200	0,200
			I	Coef.	-0,400^†^	0,200	0,200	0,200	0,400^†^	-0,400^†^
				P	0,600	0,800	0,800	0,800	0,600	0,600
		Ses.	Tar.		C2 (n = 4)					
					A1	A2	A3	A2-A1	A3-A1	A3-A2
	NPF	A	N	Coef.	0,894^†^	0,949^†^	0,600^†^	0,949^†^	-0,949^†^	-1000^†^
				p	0,106	0,051	0,400	0,051	0,051	--
			I	Coef.	0,949^†^	0,949^†^	0,738^†^	-0,775^†^	-0,316^†^	-0,775^†^
				p	0,051	0,051	0,262	0,225	0,684	0,225
		B	N	Coef.	0,894^†^	0,949^†^	0,600^†^	0,949^†^	-0,949^†^	-1000^†^
				p	0,106	0,051	0,400	0,051	0,051	--
			I	Coef.	0,949^†^	0,949^†^	0,738^†^	-0,775^†^	-0,316^†^	-0,775^†^
				p	0,051	0,051	0,262	0,225	0,684	0,225
		C	N	Coef.	0,000	0,105	-0,400	0,105	-0,632^†^	-0,400^†^
				p	> 0,999	0,895	0,600	0,895	0,368	0,600
			I	Coef.	0,316	0,316	0,316	-0,775^†^	-0,949^†^	-0,775^†^
				p	0,684	0,684	0,684	0,225	0,051	0,225
		Total	N	Coef.	0,894^†^	0,949^†^	0,600^†^	0,949^†^	-0,949^†^	-1000^†^
				p	0,106	0,051	0,400	0,051	0,051	--
			I	Coef.	0,949^†^	0,949^†^	0,738^†^	-0,775^†^	-0,316^†^	-0,775^†^
				p	0,051	0,051	0,262	0,225	0,684	0,225
	PCC	A	N	Coef.	-0,800^†^	-0,800^†^	-0,400^†^	-0,600^†^	0,600^†^	0,600^†^
				p	0,200	0,200	0,600	0,400	0,400	0,400
			I	Coef.	-1,000^†^	-1,000^†^	-0,400^†^	-0,800^†^	0,738^†^	0,949^†^
				p	--	--	0,600	0,200	0,262	0,051
		B	N	Coef.	-0,800^†^	-0,800^†^	-0,400^†^	-0,600^†^	0,600^†^	0,600^†^
				p	0,200	0,200	0,600	0,400	0,400	0,400
			I	Coef.	-1,000^†^	-1,000^†^	-0,400^†^	-0,800^†^	0,738^†^	0,949^†^
				p	--	--	0,600	0,200	0,262	0,051
		C	N	Coef.	0,200	0,200	-0,600^†^	0,400^†^	-0,400^†^	-0,400^†^
				p	0,800	0,800	0,400	0,600	0,600	0,600
			I	Coef.	-0,400^†^	-0,400^†^	-0,600^†^	-0,200	-0,105	0,316^†^
				p	0,600	0,600	0,400	0,800	0,895	0,684
		Total	N	Coef.	-0,800^†^	-0,800^†^	-0,400^†^	-0,600^†^	0,600^†^	0,600^†^
				p	0,200	0,200	0,600	0,400	0,400	0,400
			I	Coef.	-1,000^†^	-1,000^†^	-0,400^†^	-0,800^†^	0,738^†^	0,949^†^
				p	--	--	0,600	0,200	0,262	0,051
	PCC-R	A	N	Coef.	-0,600^†^	-0,600^†^	-0,800^†^	-0,600^†^	0,600^†^	0,600^†^
				p	0,400	0,400	0,200	0,400	0,400	0,400
			I	Coef.	-0,800^†^	-1,000^†^	-0,800^†^	-0,400^†^	0,738^†^	1,000^†^
				p	0,200	--	0,200	0,600	0,262	--
		B	N	Coef.	-0,600^†^	-0,600^†^	-0,800^†^	-0,600^†^	0,600^†^	0,600^†^
				p	0,400	0,400	0,200	0,400	0,400	0,400
			I	Coef.	-0,800^†^	-1,000^†^	-0,800^†^	-0,400^†^	0,738^†^	1,000^†^
				p	0,200	--	0,200	0,600	0,262	--
		C	N	Coef.	0,400^†^	0,400^†^	-0,200	0,400^†^	-0,400^†^	-0,400^†^
				p	0,600	0,600	0,800	0,600	0,600	0,600
			I	Coef.	-0,200	-0,400^†^	-0,200	-1,000^†^	-0,105	0,400^†^
				p	0,800	0,600	0,800	--	0,895	0,600
		Total	N	Coef.	-0,600^†^	-0,600^†^	-0,800^†^	-0,600^†^	0,600^†^	0,600^†^
				p	0,400	0,400	0,200	0,400	0,400	0,400
			I	Coef.	-0,800^†^	-1,000^†^	-0,800^†^	-0,400^†^	0,738^†^	1,000^†^
				p	0,200	--	0,200	0,600	0,262	--
	PDI	A	N	Coef.	0,600^†^	0,600^†^	0,949^†^	0,800^†^	0,000	-0,600^†^
				p	0,400	0,400	0,051	0,200	> 0,999	0,400
			I	Coef.	0,800^†^	1,000^†^	0,800^†^	0,800^†^	-0,600^†^	-1,000^†^
				p	0,200	--	0,200	0,200	0,400	--
		B	N	Coef.	0,600^†^	0,600^†^	0,949^†^	0,800^†^	0,000	-0,600^†^
				p	0,400	0,400	0,051	0,200	> 0,999	0,400
			I	Coef.	0,800^†^	1,000^†^	0,800^†^	0,800^†^	-0,600^†^	-1,000^†^
				p	0,200	--	0,200	0,200	0,400	--
		C	N	Coef.	-0,400^†^	0,400^†^	0,316^†^	-0,200	0,800^†^	0,400^†^
				p	0,600	0,600	0,684	0,800	0,200	0,600
			I	Coef.	0,200	0,400^†^	0,200	0,800^†^	0,400^†^	-0,400^†^
				p	0,800	0,600	0,800	0,200	0,600	0,600
		Total	N	Coef.	0,600^†^	0,600^†^	0,949^†^	0,800^†^	0,000	-0,600^†^
				P	0,400	0,400	0,051	0,200	1,000	0,400
			I	Coef.	0,800^†^	1,000^†^	0,800^†^	0,800^†^	-0,600^†^	-1,000^†^
				P	0,200	--	0,200	0,200	0,400	--
		Ses.	Tar.		Total (n = 8)					
					A1	A2	A3	A2-A1	A3-A1	A3-A2
NPF		A	N	Coef.	0,558^†^	0,518^†^	0,350^†^	0,469^†^	-0,315^†^	-0,551^†^
				p	0,151	0,188	0,395	0,241	0,447	0,157
			I	Coef.	0,501^†^	0,451^†^	0,469^†^	-0,077	0,089	-0,412^†^
				p	0,206	0,263	0,241	0,856	0,833	0,310
		B	N	Coef.	0,364^†^	0,289	0,200	0,210	-0,315^†^	-0,350^†^
				p	0,300	0,487	0,634	0,618	0,447	0,395
			I	Coef.	0,300^†^	0,300^†^	0,346^†^	-0,077	0,115	-0,247
				p	0,470	0,470	0,401	0856	0,786	0,555
		C	N	Coef.	-0,036	-0,048	-0,100	0,025	-0,101	-0,050
				p	0,932	0,910	0,814	0,954	0,812	0,906
			I	Coef.	0,050	0,150	0,025	0,077	0,000	-0,247
				p	0,906	0,723	0,954	0,856	> 0,999	0,555
		Total	N	Coef.	0,364^†^	0,289	0,200	0,210	-0,315^†^	-0,350^†^
				p	0,376	0,487	0,634	0,618	0,447	0,395
			I	Coef.	0,300^†^	0,300^†^	0,346^†^	-0,077	0,115	-0,247
				p	0,470	0,470	0,401	0,856	0,786	0,555
PCC		A	N	Coef.	-0,506^†^	-0,262	0,012	0,190	0,659^†^	0,252
				p	0,201	0,531	0,978	0,651	0,076	0,548
			I	Coef.	-0,299	-0,619^†^	-0,395^†^	-0,554^†^	-0,072	0,415^†^
				p	0,471	0,102	0,333	0,154	0,866	0,307
		B	N	Coef.	-0,313^†^	0,000	0,144	0,405^†^	0,599^†^	0,084
				p	0,450	> 0,999	0,734	0,320	0,117	0,844
			I	Coef.	-0,084	-0,476^†^	-0,240	-0,386^†^	0,072	0,390^†^
				p	0,844	0,233	0,568	0,346	0,866	0,339
		C	N	Coef.	-0,096	0,262	-0,060	0,619^†^	0,132	-0,359^†^
				p	0,820	0,531	0,888	0,102	0,756	0,382
			I	Coef.	0,012	-0,333^†^	-0,335^†^	-0,096	-0,108	0,171
				p	0,978	0,420	0,417	0,820	0,799	0,686
		Total	N	Coef.	-0,313^†^	0,000	0,144	0,405^†^	0,599^†^	0,084
				p	0,450	> 0,999	0,734	0,320	0,117	0,844
			I	Coef.	-0,084	-0,476^†^	-0,240	-0,386^†^	0,072	0,390^†^
				p	0,844	0,233	0,568	0,346	0,866	0,339
PCC-R		A	N	Coef.	-0,521^†^	-0,310^†^	-0,228	0,262	0,683^†^	0,071
				p	0,185	0,456	0,588	0,531	0,062	0,867
			I	Coef.	-0,405^†^	-0,659^†^	-0,563^†^	-0,357^†^	-0,072	0,515^†^
				p	0,320	0,076	0,146	-0,286	0,072	0,479
		B	N	Coef.	-0,364^†^	-0,095	-0,072	0,500^†^	0,623^†^	-0,119
				p	0,376	0,823	0,866	0,207	0,099	0,779
			I	Coef.	-0,238	-0,527^†^	-0,407^†^	-0,286	0,072	0,479^†^
				p	0,570	0,180	0,317	0,493	0,866	0,230
		C	N	Coef.	0,036	0,310^†^	0,156	0,667^†^	0,180	-0,452^†^
				p	0,932	0,456	0,713	0,071	0,670	0,260
			I	Coef.	0,024	-0,359^†^	-0,168	-0,476^†^	-0,108	0,323^†^
				p	0,955	0,382	0,691	0,233	0,799	0,435
		Total	N	Coef.	-0,364^†^	-0,095	-0,072	0,500^†^	0,623^†^	-0,119
				p	0,376	0,823	0,866	0,207	0,099	0,779
			I	Coef.	-0,238	-0,527^†^	-0,407^†^	-0,286	0,072	0,479^†^
				p	0,570	0,180	0,317	0,493	0,866	0,230
PDI		A	N	Coef.	0,204	0,238	0,311^†^	-0,095	-0,476^†^	-0,476^†^
				p	0,629	0,570	0,453	0,823	0,233	0,233
			I	Coef.	0,405^†^	0,619^†^	0,563^†^	0,443^†^	-0,048	-0,515^†^
				p	0,320	0,102	0,146	0,272	0,911	0,192
		B	N	Coef.	0,024	0,000	0,168	-0,357^†^	-0,429^†^	-0,286
				p	0,955	> 0,999	0,691	0,385	0,289	0,493
			I	Coef.	0,238	0,476^†^	0,407^†^	0,299	-0,143	-0,479†
				p	0,570	0,233	0,317	0,471	0,736	0,230
		C	N	Coef.	-0,359^†^	-0,357^†^	-0,060	-0,476^†^	-0,024	0,095
				p	0,382	0,385	0,888	0,233	0,955	0,823
			I	Coef.	-0,024	0,333^†^	0,204	0,419^†^	0,214	-0,323^†^
				p	0,955	0,420	0,629	0,301	0,610	0,435
		Total	N	Coef.	0,024	0,000	0,168	-0,357^†^	-0,429^†^	-0,286
				P	0,955	> 0,999	0,691	0,385	0,289	0,493
			I	Coef.	0,238	0,476^†^	0,407^†^	0,299	-0,143	-0,479^†^
				P	0,570	0,233	0,317	0,471	0,736	0,230

Teste de correlação de Spearman

† efeito médio/moderado ou maior

**Legenda:** Coef.: coeficiente de correlação; Ses.: sessão; A: Média das sessões 1, 4, 7 e 10; B: Média das sessões 2, 5, 8 e 11; C: Média das sessões 3, 6, 9 e 12; Tar.: tarefa; N: Nomeação; I: Imitação

## DISCUSSÃO

A eficácia das abordagens de intervenção no TSF tem sido discutida em alguns estudos^(9,11,40)^. Especificamente, as intervenções no TSF de causa desconhecida e do tipo fonológico são as mais estudadas^(13,17)^, dentre as quais encontra-se a abordagem dos ciclos^(23)^. Este estudo investigou a eficácia do PROCICLOS-A, em crianças com TSF com comprometimento fonológico, comparando o desempenho das crianças em três momentos distintos: antes da aplicação do programa, logo após a aplicação do programa, e seis semanas após o término do programa.

### Eficácia do PROCICLOS-A

Os resultados do estudo indicam evidências de eficácia do PROCICLOS-A, na medida que foram observados melhores resultados para cada uma das crianças, independentemente da frequência das sessões, nas avaliações pós-intervenção (A2 e A3). Na análise inferencial considerando as medidas NPF, PCC, PCC-R e PDI, nos três momentos de avaliação (A1, A2 e A3), foi possível observar diferença significante para todos, exceto para NPF na prova de Nomeação, e PCC na prova de Imitação, tanto para C1 quanto para C2. Em relação ao NPF, cabe destacar que muitas vezes um processo fonológico ainda continua presente na fala de uma criança com TSF após o primeiro ciclo de intervenção, porém a sua ocorrência diminui^(24)^. Quanto ao PCC, um fator que pode interferir é que as distorções dos sons são consideradas como erros. O tamanho do efeito observado nas várias análises reforça a evidência de eficácia do PROCICLOS-A, uma vez que os valores foram entre moderado e grande, para todos as medidas, tanto para C1 quanto para C2. Isso indica a ocorrência de um melhor desempenho das crianças após aplicação do PROCICLOS-A, independente da frequência a qual foram submetidos.

O PROCICLOS-A é um programa de intervenção composto por um ciclo de 12 sessões, no qual são trabalhados dois processos fonológicos e quatro sons alvos, preferencialmente aqueles que são estimuláveis, considerando a proposta da abordagem dos ciclos de Hodson e Paden^(23)^. Assim, a criança é exposta a novos sons antes que ocorra necessariamente o domínio do som alvo anterior^(24)^, propiciando à criança com TSF uma estimulação à tendência de generalização e eliminação dos padrões de erros de forma gradativa.

Outro destaque importante para a eficácia do PROCICLOS-A são as seis atividades desenvolvidas em todas as sessões e que trabalham diferentes habilidades, incluindo a estimulação auditiva e articulação do som alvo, bem como a utilização de pares mínimos e a consciência fonológica. Farquharson^(41)^ cita que crianças com TSF necessitam de abordagens que englobam diferentes habilidades que supram as dificuldades de cada caso e esse tipo de abordagem pode resultar em melhores resultados em relação àquelas que têm foco específico em uma só habilidade.

A estimulação de diferentes habilidades na intervenção para o TSF de causa desconhecida também foi destacada por Brosseau-Lapré e Roepke^(5)^, numa abordagem que envolve percepção auditiva e produção de fala, a qual foi eficaz para crianças com TSF. As autoras recomendam que as intervenções garantam que as crianças desenvolvam um bom conhecimento perceptivo do som alvo, forneçam prática de produção com intensidade suficiente e incorporem atividades metafonológicas durante a terapia fonoaudiológica, para promover a precisão da produção da fala no ambiente terapêutico, a generalização para a fala espontânea, além de estabelecer as bases para as habilidades de leitura.

### Análise da Influência da Dose de Frequência das Sessões

No presente estudo, um dos objetivos foi verificar a influência da frequência semanal das sessões na eficácia do PROCICLOS-A. Explorar essa questão foi motivada por ser uma dúvida comum em relação ao que é melhor para a criança com TSF. Porém, a influência da dose da frequência das sessões ainda é um tema discutido na literatura e que merece a atenção dos pesquisadores para identificar quais os componentes da intensidade de intervenção influenciam mais a eficácia das abordagens de intervenção^(9,20,22,40,42)^.

Alguns fatores podem interferir no desempenho de uma criança com TSF, durante a intervenção, independentemente da abordagem adotada. Dentre estes está a intensidade cumulativa da intervenção, proposta por Warren et al^(43)^, que pode fornecer um indicador geral da intensidade total de uma intervenção. Para calcular este índice, três componentes são importantes, o primeiro a dose de momentos de estimulação do alvo da intervenção; segundo a frequência da dose das sessões de intervenção em um período de tempo como por exemplo por semana; e a duração total da intervenção, por exemplo o número de semanas. A intensidade cumulativa da intervenção tem sido indicada como um aspecto importante para se analisar as abordagens de intervenção^(13)^. Além dos três componentes da intensidade cumulativa da intervenção, Warren et al.^(43)^ indicam que a forma da dose é um elemento importante da intervenção. Esta se refere à tarefa ou atividade típica aplicada para estimular o alvo da intervenção, sendo que a forma da dose facilita a identificação da dose executada.

A variação de cada um dos componentes da intensidade cumulativa pode ser estudada, o que permitiria identificar qual deles proporcionaria melhores resultados da intervenção. No PROCICLOS-A os quatro componentes foram estruturados sendo a forma da dose estabelecida por meio das atividades e estratégias para cada sessão, dose de estímulos na sessão em torno de 100 por 50 minutos, a frequência das sessões uma ou duas vezes por semana correspondendo a C1 ou C2, e a duração total do ciclo foi de 12 semanas para C1 e de 6 semanas para C2. Portanto, no presente estudo as variáveis controladas foram a forma da dose, a dose e a intensidade cumulativa da intervenção, as variáveis independentes à dose da frequência, e a duração total da intervenção. A intensidade cumulativa da intervenção^(43)^ é calculada considerando a dose x frequência das sessões x duração total da intervenção, o que resulta em 1200 tanto para C1 como para C2. Considerando esse conceito de intensidade da intervenção observou-se que esta foi a mesma para os dois grupos uma vez que para C1 a intervenção teve a duração de 12 semanas e para C2 a duração de seis semanas. O resultado encontrado no estudo sugere que a duração total da intervenção, em número de horas, mostrou-se mais importante para a melhora das crianças tornando a frequência de uma ou duas vezes na semana uma variável de menor impacto.

### Desempenho nas sessões do PROCICLOS-A

No PROCICLOS-A a forma da dose, ou seja, a definição das atividades aplicadas nas sessões foi previamente delineada com o intuito de atingir as habilidades que interferem na fala^(5,11,23,42)^. Além das atividades, todos os estímulos foram selecionados de acordo com os sons alvos envolvidos nos padrões de erros ou processos fonológicos mais observados em crianças com TSF falantes do português brasileiro.

Como indicado no método, para verificar o desempenho das crianças do estudo foi criado um sistema de pontuação das atividades, sendo o total esperado para cada som alvo calculado em função dos estímulos presentes nas sessões. Assim, pode-se observar a porcentagem de acertos obtidas nas sessões e especificamente em cada uma das atividades, indicando as habilidades em que houve maior ou menor dificuldade para as crianças com TSF estudadas.

Os resultados da análise do desempenho durante as sessões de intervenção, permitiu observar que as crianças com TSF tiveram bom aproveitamento das atividades que envolveram percepção auditiva, consciência fonológica, pares mínimos e treino articulatório, que são habilidades apontadas como deficitárias em crianças com TSF com comprometimento fonológico^(5,11,44)^.

O desempenho obtido pelas crianças em cada sessão não teve correlação estatisticamente significante com a melhora observada nas medidas analisadas nos momentos de avaliação (Tabela 4). Assim, independentemente do aproveitamento nas sessões, as medidas da fonologia ABFW refletiram melhora das crianças com TSF do presente estudo. Esse achado sugere que durante as sessões do PROCICLOS-A, a forma da dose, as atividades planejadas, e a dose dos estímulos possibilitaram modificações no desempenho da criança observadas na comparação das medidas da fonologia ABFW entre os momentos de avaliação (Tabela 1).

Os dados obtidos por meio da pontuação do PROCICLOS-A se mostraram úteis para entender as dificuldades e potencialidades de cada criança, uma vez que na sessão são trabalhadas diferentes habilidades, e cada atividade possui um objetivo específico. No geral, as crianças apresentaram um menor desempenho nas sessões em que lhe foram apresentados um novo som alvo e, nas sessões seguintes, foi observada uma melhora do desempenho, o que mostra o aprendizado das crianças no decorrer das três sessões em que é trabalhado um som alvo. No estudo de Rudolph e Wendt^(24)^, um dos sujeitos experenciou algo similar, quando submetido a abordagem dos ciclos de Hodson e Paden^(23)^. A criança em questão apresentou melhor desempenho na produção dos sons alvos, no decorrer do tratamento, porém, nos períodos de introdução de novos sons, apresentou nítido declínio de seu desempenho. As autoras sugerem que, apesar de a introdução de um novo som apresentar piora no desempenho, a criança mostrava melhora no decorrer do atendimento, indicando um aprendizado e posterior estabilização de novo som alvo no inventário fonológico da criança.

Dentre as limitações do presente estudo destaca-se o pequeno número de crianças em cada grupo que é um fator importante para confirmar a eficácia da abordagem PROCICLOS-A em função da dose da frequência das sessões. Ainda, é importante realizar mais pesquisas que correlacionam a dose de estímulos e sua relação com a dose da frequência das sessões, para entender melhor o impacto de cada uma dessas variáveis na intervenção para o TSF.

## CONCLUSÃO

O estudo realizado mostrou evidências da eficácia do PROCICLOS-A na medida que todas as crianças melhoraram, independentemente dos valores iniciais das medidas analisadas na fonologia ABFW e, o tamanho do efeito foi de médio a grande entre os momentos de avaliação. Em relação à dose da frequência das sessões, o estudo indica que o número total de horas de intervenção parece interferir mais do que o número de sessões por semana, quando foi mantido a intensidade cumulativa da intervenção.

O estudo também apresentou resultados que mostram a melhora no desempenho de cada criança no decorrer do processo terapêutico, independentemente do som alvo. Isso indica que as atividades do PROCICLOS-A contribuem para o aprendizado de cada som de forma gradativa em cada sessão, independentemente da gravidade no início da intervenção.

Estes primeiros resultados obtidos evidenciam que a proposta de intervenção PROCICLOS-A é promissora para o tratamento de crianças com TSF idiopático do tipo fonológico.
